# Explicit instructions and consolidation promote rewiring of automatic behaviors in the human mind

**DOI:** 10.1038/s41598-017-04500-3

**Published:** 2017-06-29

**Authors:** Emese Szegedi-Hallgató, Karolina Janacsek, Teodóra Vékony, Lia Andrea Tasi, Leila Kerepes, Emőke Adrienn Hompoth, Anna Bálint, Dezső Németh

**Affiliations:** 1 Institute of Psychology, University of Szeged, Egyetem utca 2, H–6722 Szeged, Hungary; 2 Institute of Psychology, Eötvös Loránd University, Izabella utca 46, H–1064 Budapest, Hungary; 3 MTA-ELTE NAP B Brain, Memory and Language Research Group, Institute of Cognitive Neuroscience and Psychology, Research Centre for Natural Sciences, Hungarian Academy of Sciences, Magyar tudósok körútja 2, H–1117 Budapest, Hungary; 4 Department of Neurology, University of Szeged, Semmelweis u. 6, H-6725 Szeged, Hungary; 5 Department of Emergency Medicine, University of Szeged, Semmelweis u. 6, H-6725 Szeged, Hungary; 6 Department of Child and Adolescent Psychiatry, University of Szeged, Boldogasszony sgt.15., 6725 Szeged, Hungary

## Abstract

One major challenge in human behavior and brain sciences is to understand how we can rewire already existing perceptual, motor, cognitive, and social skills or habits. Here we aimed to characterize one aspect of rewiring, namely, how we can update our knowledge of sequential/statistical regularities when they change. The dynamics of rewiring was explored from learning to consolidation using a unique experimental design which is suitable to capture the effect of implicit and explicit processing and the proactive and retroactive interference. Our results indicate that humans can rewire their knowledge of such regularities incidentally, and consolidation has a critical role in this process. Moreover, old and new knowledge can coexist, leading to effective adaptivity of the human mind in the changing environment, although the execution of the recently acquired knowledge may be more fluent than the execution of the previously learned one. These findings can contribute to a better understanding of the cognitive processes underlying behavior change, and can provide insights into how we can boost behavior change in various contexts, such as sports, educational settings or psychotherapy.

## Introduction

French drivers face a real challenge when they have to drive in England for the first time. They might look in the wrong direction when checking the traffic, and incorrectly assume that there is no other vehicle, so they are free to go ahead at a crossroad. Their already well-developed perceptual-motor skill of driving becomes ineffectual or even harmful by leading them to false predictions in the new environment. Our study can contribute to a better understanding of how we can rewire our perceptual-motor skills in such situations. Can we adapt to the changed regularities of the environment without any external help, purely implicitly?

The aim of our study was to test, in a controlled experimental setting, how we can update – *rewire* – our knowledge of sequential/statistical regularities that thought to be an essential aspect of many everyday skills, such as playing a musical instrument or video games^1^, learning/processing languages^2, 3^, and social skills^4, 5^. Modification of such knowledge can be empirically tested by teaching two differing sets of regularities (i.e., sequences) to participants. Characterizing the interference between the first-learned sequence and the newly encountered one is the key to understand the rewiring process. Retroactive interference can hinder our ability to activate old sequence knowledge once new reguralities have been learned^6–10^. Likewise, our previously developed automatisms can make it more difficult to learn new regularities, termed as proactive interference. Proactive interference effects were found even after limited practice when sequence A and sequence B were highly dissimilar^7^; and it was also detected by contrasting performance on those chunks of movements that were common to both sequences with those that differed^11^. While retroactive interference can be beneficial for overwriting old knowledge of sequential regularities, proactive interference works against successful rewiring.

**Figure 1:**
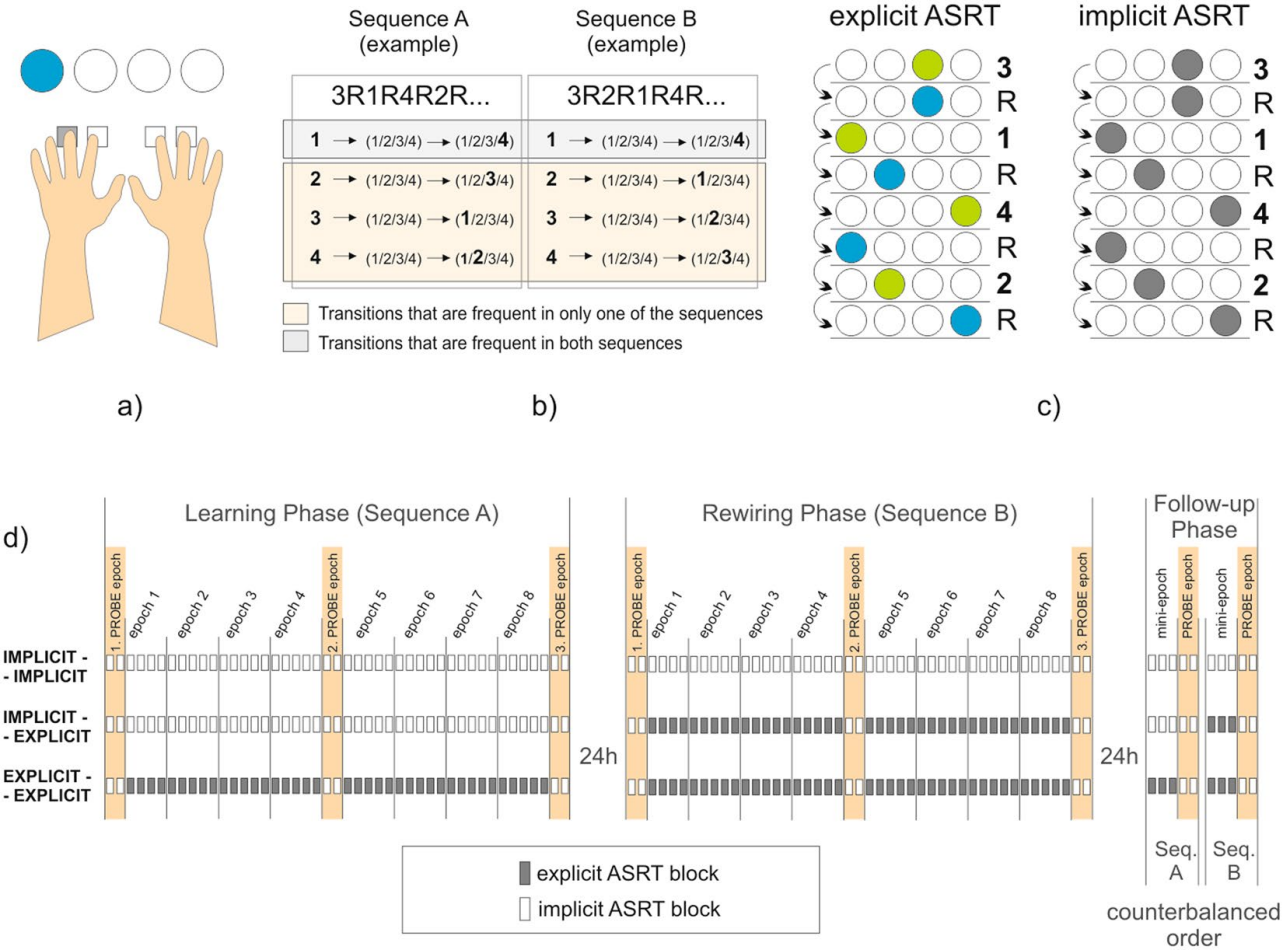
Methods and procedure. (**a**) ASRT task: participants were asked to respond to stimuli appearing on one of four locations, and press the corresponding key as fast and as accurately as they could. (**b**) Examples of sequences and their shared/differing transitions. Numbers indicate the locations of stimuli (1, 2, 3 or 4). The notation “R” indicates a random location out of the four possible ones. Knowing what stimulus appeared two trials before enables participants to anticipate the next stimulus as there is always a highly probable continuation (highlighted) and three less probable continuations. In Sequence A, for example, two trials after encountering a stimulus on the 2nd location, the most probable stimulus is one on the 3rd location (all the remaining possibilities being equally less likely). Sequence A and Sequence B share some of their transitional probabilities (e.g. encountering stimulus on the 4th location two trials after encountering one on the 1st location); and they differ on others (e.g. encountering stimulus on the 3rd location predicts a stimulus on the 2nd location two trials later in Sequence B, but not in Sequence A). Interference effects could be detected by contrasting the magnitude of learning of changed and unchanged sequence parts. (**c**) In the implicit version of the task, random and pattern trials appeared in the same color, thus they were indistinguishable to participants. In the explicit version of the task, random and pattern trials were of different colors, and participants were asked to keep track of the repeating pattern. (**d**) There were 85 trials in a block. There were 45 blocks in the Learning and Rewiring Phase, collapsed into bigger sections (epochs) for analysis. Probe epochs were implicit for all participants. Here we focus on experimental epochs (probe epochs led to similar results, for details see Supplementary Results 2.3–2.4). In the Follow-up Phase, we aimed to test participants’ performance on both sequences after equal amount of practice on each, without introducing much relearning. Therefore only 5 + 5 blocks of both sequences were presented on the third day. Two of these blocks were probe blocks.

To date no study has focused on the entire process of rewiring in humans that captured both retroactive and proactive interference effects, and at the same time assessed the impact of explicit processes in one experimental design. Here we present a study with such a design to tackle the question whether we can overcome the negative consequences of the interference without conscious effort (i.e., implicitly) or the awareness about the changed reguralities (i.e., explicitness) is essential for rewiring. Eighty-four healthy young adults performed a four-choice reaction time task (Fig. 1a) on three consecutive days. The presentation order of the stimuli followed a probabilistic sequence on the first day (Sequence A in the Learning Phase), then this sequence changed to a different one on the second day (Sequence B in the Rewiring Phase). The two sequences shared some of their transitional probabilities (for details see Methods and Fig. 1b), meaning that at some points in Sequence B the most probable upcoming stimulus was the same as in Sequence A (*unchanged* sequence parts). Other transitional probabilities changed: the most probable continuation of the previous trials was different from that on the previous day (*changed* sequence parts). This way we could compare learning with and without interference from the previous day (more details in Supplementary Methods 1.2). In addition, to assess the impact of explicit information processing, sequences were learned either explicitly or implicitly: implicit learners were not aware of the sequence structures, while explicit learners were provided with cues that could be used on half of the trials (Fig. 1c). We hypothesized that – if explicitness does help rewiring – it should be expected that explicit learners perform better even on those trials on which they did not have any explicit advantage over the implicit learners. Three groups of participants were compared: the Implicit-Implicit group learned both sequences implicitly (thus rewiring was also implicit); the Implicit-Explicit group learned the first sequence implicitly and the second sequence explicitly; the Explicit-Explicit group learned both sequences explicitly (Fig. 1d).

**Figure 2:**
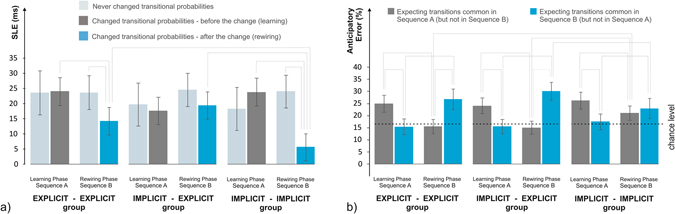
Learning and Rewiring. (**a**) Statistical learning effect (SLE) in the Learning and Rewiring Phase. The magnitude of SLE indicates the difference of reaction times (RTs) given to frequent transitions (more probable stimuli) in contrast to infrequent transitions (less probable stimuli). Some of the transitions had constant frequency in the Learning Phase and Rewiring Phase (unchanged transitions, light grey bars), while other transitions swapped their frequency – previously infrequent transitions became frequent in the Rewiring Phase, and vice versa (changed transitions﻿, dark grey bars – before the change, blue bars – after the change occurred ﻿). Adapting to the changed statistical structure in the Rewiring Phase was shown to be more difficult than learning the contingencies in the first place in the Learning Phase. This was shown by SLEs being – on average – smaller for the changed transitions after the change in frequencies took place in the Rewiring Phase (blue bars) than before the change (dark grey bars). The Implicit-Explicit group did not show signs of such difficulty. (**b**) When a less probable stimulus came up, participants sometimes erroneously pressed the key corresponding to the most probable stimulus, termed as anticipatory errors. As two (partly) different sequences were taught, we differentiated between anticipations of Sequence A’s most probable stimuli, and that of Sequence B’s. Percentage of anticipatory errors of Sequence A (learned in the Learning Phase, grey bars) and Sequence B (learned in the Rewiring Phase, blue bars) over the two Phases, and chance level for anticipatory errors (dotted line) are shown. Each group showed adaptation to the current sequence, as anticipations for Sequence A were above chance level in the Learning Phase, while anticipations of Sequence B were above chance level in the Rewiring Phase. The Implicit-Implicit group additionaly showed above chance level anticipations of Sequence A during the Rewiring Phase, indicating the continuing influence of their knowledge gained in the Learning Phase. The solid lines connecting the bars indicate significant differences (*p* < 0.05). Error bars represent 95% confidence intervals (CIs).

We measured statistical learning as (**a**) difference in reaction times (RTs) given to anticipated (probable) stimuli in contrast to unexpected (less probable) stimuli, termed as Statistical Learning Effect (SLE), and (**b**) by determining whether erroneous responses reflect anticipations of the most probable stimuli in cases when less probable trials came up (more details in Supplementary Methods 1.4–1.5). Statistical learning measured by the SLE score was evident in both the Learning and Rewiring Phase (see 95% confidence intervals, CIs, on Fig. 2a). In the Learning Phase, there could not possibly be any interference effects as only Sequence A had been introduced yet, we nevertheless contrasted the magnitude of learning of those transitional probabilities that were common during both Phases (unchanged sequence parts) and those that were about to change in the Rewiring Phase (changed sequence parts). As expected, there was no difference between the two (*p* = 0.568, Cohen’s *d* = 0.080), indicating that they were equally easy to learn (Fig. 2a, light vs. dark grey bars). In the Rewiring Phase, however, we found smaller statistical learning for the changed sequence parts (*p* < 0.001, *d* = 0.829) compared to the unchanged sequence parts (Fig. 2a, light grey vs. blue bars). This was apparent in the Implicit-Implicit (*p* < 0.001, *d* = 1.425) and Explicit-Explicit groups (*p* = 0.008, *d* = 0.737), but not in the Implicit-Explicit group (*p* = 0.128, *d* = 0.406). These results suggest that the Implicit-Explicit group was the most successful in adapting to the new statistical regularities, even to the extent that their rewired knowledge was not much different than that of the unchanged sequence parts. Interference effects were most clearly shown by contrasting the magnitude of learning of the same transitional types (changed vs. unchanged) over the two Phases. As expected, learning of the unchanged sequence parts did not decline in the Rewiring Phase (*p* = 0.151, *d* = 0.021, Fig. 2a, light grey bars in the Learning vs. Rewiring Phase), while learning of the changed sequence parts was significantly lower in Rewiring Phase than the original learning in the Learning Phase (*p* < 0.001, *d* = 0.729, Fig. 2a dark grey vs. blue bars). This pattern was apparent in the Implicit-Implicit (*p* < 0.001, *d* = 1.562) and Explicit-Explicit (*p* = 0.028, *d* = 0.850) groups, but not in the Im*p*licit-Explicit group (*p* = 0.561, *d* = 0.155) – indicating, again, that the Implicit-Explicit group was the most successful in adapting to the changes in the sequence structure. The least successful group was the Implicit-Implicit group, evidenced by their statistical knowledge of the changed sequence parts in the Rewiring Phase (Fig. 2a, blue bars) being significantly smaller than both the Explicit-Explicit (*p* = 0.028, *d* = 0.743) and Implicit-Explicit groups’ (*p* < 0.001, *d* = 1.198). Additional analysis of the time course of rewiring revealed that the Implicit-Implicit group showed significant learning of the changed transitional probabilities only in the second half of the Rewiring Phase, suggesting slower updating of the previously acquired statistical knowledge (i.e., larger proactive interference) compared to the other two groups (for more details see Supplementary Results 2.1.1).

On some of the trials, participants pressed a key that did not correspond to the stimulus. Some of these errors reflected anticipations of the most probable stimulus when the actual stimulus was a less probable one, termed as *anticipatory errors*. As two sequences were taught, we can measure anticipations of Sequence A’s most probable transitions and those of Sequence B’s (errors that could be regarded as anticipations of both sequences were not analysed). We compared the proportion of anticipatory errors to each other (anticipations of Sequence A vs. anticipations of Sequence B), and to a baseline proportion that could be expected by chance (16.67%, see Fig. 2b). As expected, the Learning Phase was dominated by anticipations of Sequence A (dark grey bars on Fig. 2b), while the Rewiring phase was dominated by anticipations of Sequence B (both *p* < 0.001, both *d* > 1.061, blue bars on Fig. 2b). From another point of view, there were less anticipations of Sequence B in the Learning Phase than in the Rewiring Phase, and vice versa (both *p* < 0.001, both *d* > 0.979). This pattern of results was observed in all groups, although effect sizes were substantially smaller in the the Implicit-Implicit group (both *d* < 0.672) than in the other groups (all *d* > 1.226). Most importantly, anticipations of Sequence B in the Rewiring Phase (that indicate adaptation to the new sequence structure) were less pronounced in the Implicit-Implicit group than in the Implicit-Explicit group (*p* = 0.047, *d* = 0.721), while anticipations of Sequence A in the same Phase (indicating the continuing influence of the knowledge gained in the Learning Phase) were more pronounced in the Implicit-Implicit group than in the Implicit-Explicit and the Explicit-Explicit groups (both *p* < 0.036, both *d* > 0.795). The Implicit-Implicit group showed no significant difference in proportions of anticipating Sequence A and Sequence B during the Rewiring Phase (*p* = 0.529, *d* = 0.225), both being above chance level (see 95% CIs on Fig. 2b). These results clearly point to the continuing influence of the no-longer valid statistical knowledge gained in the Learning Phase – that is, proactive interference – in the Implicit-Implicit group (see also Supplementary Results 2.1.2). The remaining two groups who performed the Rewiring Phase explicitly showed no signs of such interference, indicating a beneficial effect of awareness about the sequence structures.

**Figure 3:**
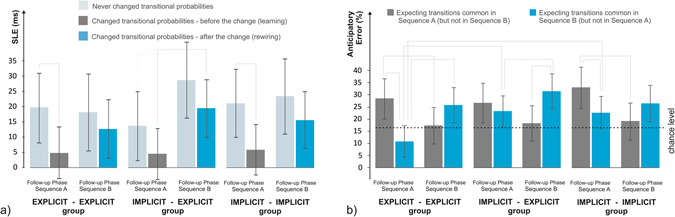
Testing the efficiency of the rewiring process after a 24-hour consolidation period, in the Follow-up Phase. (**a**) Overall, SLEs for the changed sequence parts (dark grey and blue bars) were smaller than that of the unchanged sequence parts (light grey bars). In the case of the changed sequence parts, statistical structure that corresponded to Sequence B (that was learned in the Rewiring Phase) was retained better than statistical structure that corresponded to Sequence A (that was learned in the Learning Phase) – the latter not reliably differing from zero (as shown by 95% CIs). This pattern indicates adaptation to the changed statistical regularities of Sequence B taking place in the Rewiring Phase, and no observable proactive interference of Sequence A, thus, overall successful rewiring. No group differences were observed. (**b**) Chance level for anticipatory errors are shown by the dotted line. Each group showed adaptation to the current sequence as anticipations for Sequence A were above chance level when performing Sequence A in the Follow-up Phase; while anticipations for Sequence B were above chance level when performing Sequence B. This pattern suggests that the old and new knowledge, acquired on the first and second day of the experiment, coexisted after a 24-hour delay period, and were accessible when required. The solid lines connecting the bars indicate significant differences (*p* < 0.05). Dotted lines indicate trend level differences (*p* < 0.10). Error bars represent 95% CIs.

Participants were retested on the third day (in the Follow-up Phase, Fig. 1d) for both sequences to test whether the first one became overwritten by the second one. Participants showed better performance on the transitions that were frequent in both the Learning and Rewiring Phases (Fig. 3a, light grey bars) than on those that were frequent in only one of the Phases (Fig. 3a, dark grey and blue bars; *p* = 0.003, *d* = 0.506), which is not surprising given that the former ones were practiced almost twice as much. More importantly, better performance was expressed for Sequence B than for Sequence A (*p* = 0.015, *d* = 0.404). This pattern of results may indicate that statistical knowledge for Sequence A became partly overwritten by knowledge of Sequence B, showing retroactive interference which is beneficial for the rewiring process. No group differences were observed (*p*s > 0.303; see also Supplementary Results 2.2.1).

When performing Sequence A on the third day of the study, anticipations of Sequence A were more common than anticipations of Sequence B (*p* = 0.004, *d* = 0.533), and than what might have been expected by chance (Fig. 3b, dark grey bars). When performing Sequence B, on the other hand, anticipations of Sequence B outnumbered anticipations of Sequence A (*p* = 0.009, *d* = 0.503), and were more numerous than expected by chance (Fig. 3b, blue bars). From another point of view, anticipations of Sequence A were significantly more pronounced when performing Sequence A than when performing Sequence B, and vice versa (both *p* < 0.003, *d* > 0.494). This pattern of results indicate no proactive or retroactive interference effects, as participants were able to quickly adapt to changes in the statistical structure, and suggests that knowledge about the two statistical structures coexist and can be adaptively used in the appropriate situation. No group differences were observed (*p* = 0.745; see also Supplementary Results 2.2.2).

In our study, we compared performance of groups that learned/rewired their knowledge with vs. without explicit cues. The Free Generation and the Triplet Sorting Tasks were used to explore to what extent their knowledge remained implicit or became explicit (see Supplementary Methods 1.6.1–2). In the Free Generation Task, they were asked to generate alternating sequences similar/dissimilar to the ones they encountered during the experiment. The results revealed clear group differences with participants in the explicit conditions exhibiting better performance, as a result of the explicit cues and instructions. Participants who learned/rewired their knowledge without explicit cues (i.e., implicitly) might have also gained some explicit knowledge about the regularities as well, although we cannot rule out the possibility that they used different strategies during task compared to the explicit group, and those strategies resulted in a somewhat similar performance in the end (for details see Supplementary Results 2.5.1). The Triplet Sorting Task more directly tested their knowledge about the same statistical structures (triplets) that also provided the basis of the RT and anticipatory error analyses. The results of this task support the interpretation that knowledge of these statistical structures remained implicit for the implicit learners (Supplementary Results 2.5.2). This interpretation is also in line with previous ASRT studies showing that participants remain unaware of the stimulus structure if it is not explicitly cued^12^, and even after extended practice (e.g., ten days^13^). Nevertheless, we can never totally exclude the possibility that some degree of explicit knowledge developed even if the sequence structure was not cued.

In summary, we found successful rewiring of the acquired knowledge in all three experimental groups. In the Rewiring Phase the group that learned implicitly and rewired with the help of explicit cues (i.e., the Implicit-Explicit group) showed better performance than the other groups. In other words, explicit cues during the rewiring process led to faster adaptation to the changed reguralities, evidenced both in the cued part of the task (experimental epochs) as well as in the uncued part (probe epochs, see Supplementary Results 2.3). By the end of the rewiring period, all groups showed similar performance suggesting an efficient but slower rewiring in the Implicit-Implicit group as well. We also found evidence that the first learned sequence was accessible when needed, shown by sequence specific anticipatory errors in the Follow-up Phase, although the motor execution of it was not as fluent as the execution of the secondly learned sequence.

The aim of our study was to test, in a controlled experimental setting, how we can update – *rewire* – our knowledge of sequential/statistical regularities that thought to be an essential aspect of many everyday skills, such as playing a musical instrument or video games^1^, learning/processing languages^2, 3^, and social skills^4, 5^. Nevertheless, these skills are far more complex than the task employed in the current study, and they may encompass other types of sequential/statistical knowledge, have longer/more variable “practice schedule”, and may combine different regularities coming from different domains (such perceptual and motor). Although in our study participants were presented with regularities both in the perceptual and motor domains (correlated visual and motor stimulus streams), here we did not aim to separate the knowledge acquired in these two domains. Previous ASRT studies have showed that participants acquire both the perceptual and motor knowledge and they also retain their knowledge after a delay period^14–16^. Future studies need to directly test how various types and complexity of sequential/statistical knowledge can be updated when the underlying regularities change, whether and how sequence complexity interacts with the explicit advantage observed in our study, and whether and how these processes differ across domains.

Based on our findings, rewiring of a relatively simple statistical knowledge – that we tested in the current study – show a complex picture: proactive interference, which works against the adaptation to the changed regularities, is stronger when learning and rewiring is implicit, while explicit cues about these changed regularities can help speed up the rewiring process. After a 24-hour delay period, proactive interference is volumed down, while retroactive interference is volumed up, suggesting that consolidation of the updated knowledge about the changed regularities has a critical role in successful rewiring. The fact that both the old and new, updated knowledge seems to remain accessible highlights the adaptive nature of the human mind, making it possible to dynamically use the appropriate procedures corresponding to various environments. Our findings can contribute to a better understanding of the cognitive processes underlying behavior change.

## Methods

### Participants

Eighty-four healthy young adults took part in the experiment. Participants were recruited at University of Szeged and were randomly assigned to one of three groups: the Implicit-Implicit group (*N* = 28; 20 females; Age: *M* = 20.46 years, *SD* = 2.10), the Implicit-Explicit group (*N* = 28; 17 females; Age: *M* = 22.14 years, *SD* = 1.96), and the Explicit-Explicit group (*N* = 27; 18 females; Age: *M* = 22.54 years, *SD* = 3.33). One participant was excluded from the analysis because errorneously the same sequence was administered to him on each day of the study. Three groups did not differ in their scores on standard working memory and executive function tests (Digit Span: *p* = 0.443, *η*_*p*_^*2*^ = 0.021; Counting Span: *p* = 0.440, *η*_*p*_^*2*^ = 0.022; perseveration rates on the Wisconsin Card Sorting Task: *p* = 0.710, *η*_*p*_^*2*^ = 0.010; Stroop Test: *p* = 0.578, *η*_*p*_^*2*^ = 0.015). Participants did not suffer from any psychiatric or neurological disorders. None of the participants were aware of the purpose of the experiment. Prior to their inclusion in the study, participants provided informed consent to the procedure as approved by the research ethics committee of University of Szeged, Szeged, Hungary. The study was conducted in accordance with the Declaration of Helsinki and participants received course credits for taking part in the experiment.

### Tasks and Procedure

Participants performed a modified version of the Alternating Serial Reaction Time (ASRT) task^17^. The program was coded in Psychopy^18^. In the *Implicit variant* of the task, four light grey circles were arranged horizontally on the screen. Four buttons on the keyboard corresponded to the four locations on the screen: Z, C, B, and M, respectively. Intervening buttons were removed to minimize false buttonpresses. Participants used their left and right middle and index fingers to respond to the targets. The stimulus stayed on the screen until a correct buttonpress was made (but it remained on the screen after an unapt button was pressed). Response to stimulus interval (RSI) was set to 120 ms. One block of trials consisted of five random (preparatory) trials followed by ten repetitions of a probabilistic sequence (10 × 8 trials). After each block, there was a pause of at least 10 seconds (terminated by participants) during which the average reaction time of correct buttonpresses and the percentage of erroneous buttonpresses were displayed. Unbeknownst to the participants, the ASRT sequence consisted of a four-elements-long sequential pattern (e.g., 3–1–4–2) intersparsed with random elements (3–R–1–R–4–R–2–R). Because of the alternating pattern and random trials, the ASRT sequence can be regarded as a second order probabilistic sequence, meaning that the identity of the upcoming stimulus can be anticipated based on the *n-2* trial (one of the four possibilities is always more probable than the remaining three), regardless of the stimulus being a random or a pattern element (for more details see Supplementary Methods 1.1).

In the *Explicit variants*, participants performed a cued version of the ASRT task^19–21^. This version differed from the implicit task in three respects: firstly, random and pattern stimuli appeared in different colors (pattern elements appeared green while random elements appeared blue). Secondly, participants were instructed to pay attention primarily to the four-element long pattern (the green trials) to be able to report it after each block. Also they were told to constantly monitor this pattern and report if it changed during the course of a block (actually it never changed within a given block). Finally, the feedback after the blocks did not contain information about RTs and erroneous buttonpresses on random trials as the instruction highlighted performance on the pattern trials.

After learning an ASRT sequence (referred to as Sequence A) on the first day of the study (in the *Learning Phase*), participants were given a different ASRT sequence (referred to as Sequence B) on the second day (in the *Rewiring Phase*). Twelve different sequence combinations were used in the experiment in a counterbalanced order. All these combinations are presented in Supplementary Methods 1.3. Three groups were compared based on the instructions they received each day: The *Implicit-Implicit* group performed the implicit version of the task in both the *Learning Phase* and in the *Rewiring Phase*; the *Implicit-Explicit* group performed the implicit version in the *Learning Phase* and the explicit version in the *Rewiring Phase;* finally, the *Explicit-Explicit* group performed the explicit version in both phases.

On the third day of the study – in the *Follow-up Phase* – the magnitude of statistical knowledge for both sequences was assessed to investigate possible retroactive interference effects after a 24-hour consolidation period. Then the *Triplet Sorting Task* and the *Free Generation Task* were administered to assess the amount of explicit knowledge participants gained about both sequences (see descriptions of the tasks in Supplementary Methods 1.6). The analysis of these tasks showed that participants indeed gained more explicit knowledge about the regularities when they performed the explicit version of the task, and that the explicit cues indeed can help differentiate between the two sequences and use the acquired knowledge more appropriately in the relevant context (for details see Supplementary Results 2.5).

Finally, working memory and executive functions were also assessed on the third day. We wanted to ensure that the three groups had similar performance on these general cognitive functions, and thus the obtained results of rewiring could not be attributed to differences in these functions (see also the Participants section). The experiment took place in a quiet laboratory at University of Szeged (one participant a time). The whole procedure lasted approximately 60 minutes on the first and second day of the experiment, and an additional 40 minutes on the third day.

## Electronic supplementary material

Supplementary materials
