# Supplementary material for: Explicit instructions and consolidation promote rewiring of automatic behaviors in the human mind
Source: Sci Rep. 2017 Jun 29;7:4365. doi: 10.1038/s41598-017-04500-3 (PMC5491510; doi:10.1038/s41598-017-04500-3)
Supplement: Supplementary file 1 — Supplementary materials [file 41598_2017_4500_MOESM1_ESM.doc]

**Supplementary materials**

**Explicit instructions and consolidation promote rewiring of automatic behaviors in the human mind**

Emese Szegedi-Hallgató^*^, Karolina Janacsek^*^, Teodóra Vékony, Lia Andrea Tasi, Leila Kerepes, Emőke Adrienn Hompoth, Anna Bálint, Dezső Németh^*^

* These authors contributed equally to the paper.

**Table of contents**

[1. SUPPLEMENTARY METHODS 2](#__RefHeading___Toc477632048)

[1.1 The structure of the ASRT sequences 2](#__RefHeading___Toc477632049)

[1.2 Unchanged and changed transitions across the Learning and Rewiring Phase 4](#__RefHeading___Toc477632050)

[1.3 Sequence combinations used in the current study 6](#__RefHeading___Toc477632051)

[1.4 Calculation of Statistical Learning Effect (SLE) 8](#__RefHeading___Toc477632052)

[1.5 Calculation of anticipatory errors 9](#__RefHeading___Toc477632053)

[1.6 Tests for assessing the explicit knowledge about the sequence structures 11](#__RefHeading___Toc477632054)

[1.6.1 Free Generation Task 11](#__RefHeading___Toc477632055)

[1.6.2 Triplet Sorting Task 13](#__RefHeading___Toc477632056)

[1.7 Statistical analysis 13](#__RefHeading___Toc477632057)

[2. SUPPLEMENTARY RESULTS 14](#__RefHeading___Toc477632058)

[2.1 Dynamics of the rewiring process in the experimental epochs compared across the Learning and Rewiring Phase 14](#__RefHeading___Toc477632059)

[2.1.1 Statistical Learning Effect (SLE) 14](#__RefHeading___Toc477632060)

[2.1.2 Anticipatory Errors 17](#__RefHeading___Toc477632061)

[2.2 Testing the efficiency of the rewiring process in the experimental epochs of the Follow-up Phase 18](#__RefHeading___Toc477632062)

[2.2.1 Statistical Learning Effect (SLE) 19](#__RefHeading___Toc477632063)

[2.2.2 Anticipatory errors 19](#__RefHeading___Toc477632064)

[2.3 Dynamics of the rewiring process in the probe epochs compared across the Learning and Rewiring Phase 21](#__RefHeading___Toc477632065)

[2.3.1 Statistical Learning Effect (SLE) 21](#__RefHeading___Toc477632066)

[2.3.2 Anticipatory errors 24](#__RefHeading___Toc477632067)

[2.4 Testing the efficiency of the rewiring process in the probe epochs of the Follow-up Phase 25](#__RefHeading___Toc477632068)

[2.4.1 Statistical Learning Effect (SLE) 25](#__RefHeading___Toc477632069)

[2.4.2 Anticipatory errors 26](#__RefHeading___Toc477632070)

[2.5 Testing the explicit knowledge acquired about the sequence structures 28](#__RefHeading___Toc477632071)

[2.5.1 Free Generation Task 28](#__RefHeading___Toc477632072)

[2.5.2 Triplet Sorting Task 31](#__RefHeading___Toc477632073)

[3. REFERENCES 34](#__RefHeading___Toc477632074)

# 1. SUPPLEMENTARY METHODS

## 1.1 The structure of the ASRT sequences

In the ASRT task[^1^](#_ENREF_1) the probability of each stimulus location out of the four possible ones (0^th^ order probability) is equal (25%). For any stimulus *n*, the previous *n* – 1 trial (1^st^ order transitional probability) has no predictive value either (all pairs of stimuli are equally probable). The ASRT sequence is a 2^nd^ order probabilistic sequence because for any trial *n* there is a very probable and three less probable continuations of the sequence based on the *n – 2^th^* trial. The probabilities add up the following way: if a pattern trial comes up, the identity of this trial can be inferred with 100% certainty based on the previous pattern trial which occurred two trials before (thus 50% of all trials are predetermined as 50% of all trials are pattern trials). For example, in the case of a sequence such as 3-R-1-R-4-R-2-R, if the previous pattern trial was on the 3^rd^ location, the next pattern trial is going to be on the 1^st^. If a random trial comes up, on the other hand, it could be any of the four possible stimuli with 25% probability (irrespectively of the stimulus that occurred two trials before) – as this 25% refers to random trials only, which makes up 50% of all trials, a particular outcome has an overall probability of 12.5% in this case. Taken together, there is always a probable outcome regarding the upcoming stimulus (50% + 12.5% = 62.5%) and three less probable outcomes (12.5% each) based on trial *n–2* (Fig. S1). In the conventional (implicit) ASRT task individuals have no clue about the alternating nature of the random and pattern trials, they nevertheless learn that it is highly probable that they are going to encounter a stimulus on the 1st location if they encountered a stimulus on the 3rd location two trials before. Participants use this statistical knowledge on random and pattern trials alike.


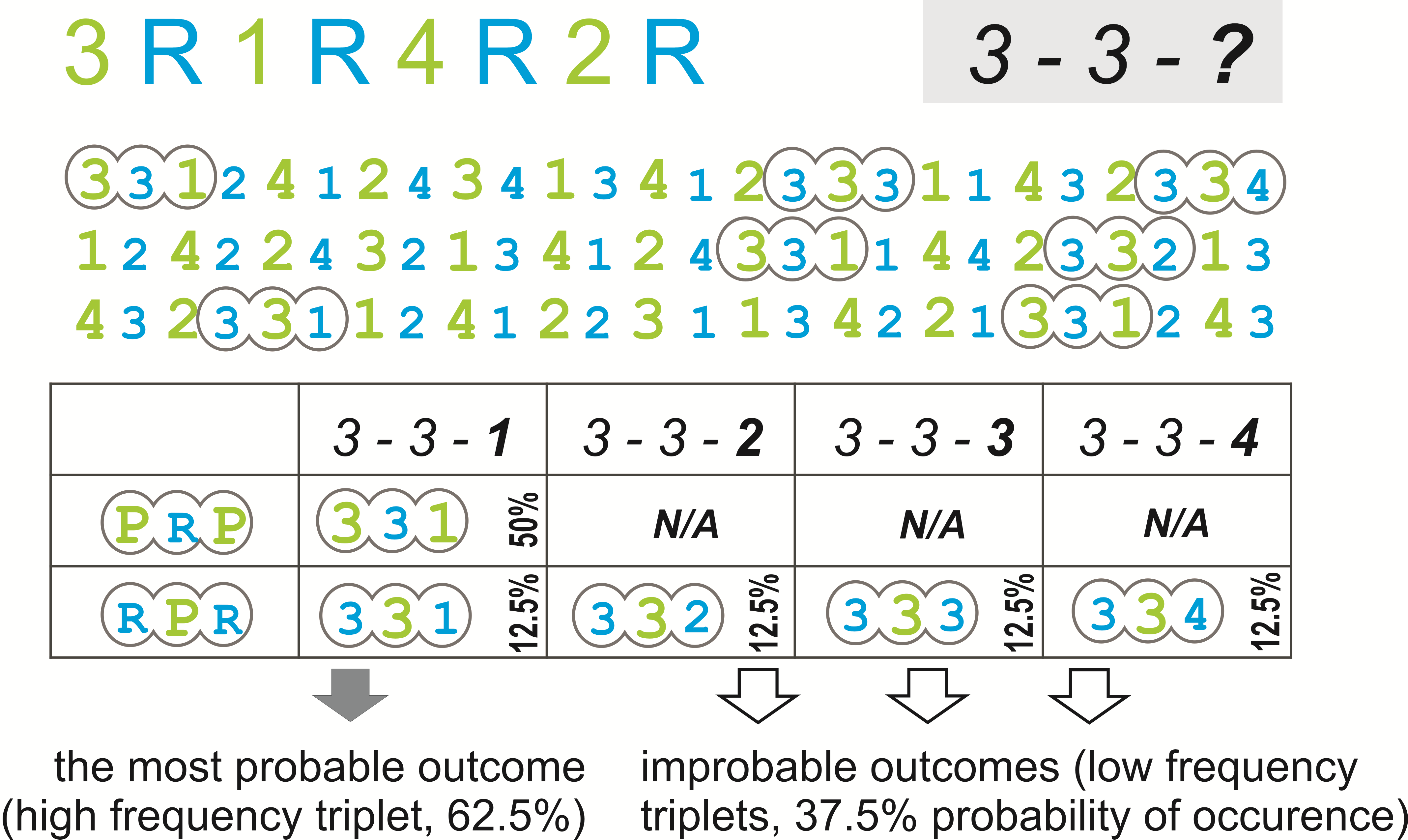


**Figure S1. The statistical structure of the ASRT sequence.** As a result of the alternation of pattern (green) and random („R”, blue) trials, there are frequent (more probable) and infrequent (less probable) combinations of three consecutive stimuli. Whatever the first two elements are of such a combination (a so called triplet), there is always a probable continuation which occurs 62.5% of the time, and three less probable continuations with a probability of 12.5% each. Frequent combinations are called *high frequency triplets*, while the infrequent combinations are called *low frequency triplets*. In the above example after encountering two consecutive stimuli on the 3^rd^ location, the most probable upcoming stimulus is one on the 1^st^ location: if the upcoming stimulus is a pattern element (green), then it will be on the 1^st^ location because of the embedded pattern. As only 50% of trials are pattern elements, there is 50% chance that the following stimulus will appear on the 1^st^ location. However, there is another 50% chance that the upcoming stimulus is a random element (green). In this case each outcome is equally likely (12.5% each). Taken together, there is 50+12.5 = 62.5% chance that the upcoming stimulus will be on the 1^st^ location – making this the most probable outcome. The comparison of high vs. low frequency triplets captures the 2^nd^ order transitional probabilities embedded in the ASRT sequence. In the original (implicit) version of the ASRT pattern and random elements are shown in the same color, and participants are not told about the embedded regularity.

If the predicted stimulus comes up, we can categorize that stimulus as the final stimulus of a so called high frequency triplet, while the less probable continuations are categorized as the final stimulus of a low frequency triplet. Thus, when we use the terms *high frequency triplet* and *low frequency triplet*, we refer to the predictability of the final element of that triplet, and we quantify participants’ suppositions as reaction times (RTs) to these final elements of triplets as a function of their probability (each element is categorized this way; the 3^rd^ element of a triplet is also a second element of the following triplet, and so on). The typical result is shorter RTs to the last elements of high frequency triplets than to the last elements of low frequency triplets.

## 1.2 Unchanged and changed transitions across the Learning and Rewiring Phase

As each participant encountered two different sequences on the subsequent days of the experiment, the same stimuli could be a probable continuation of the same contexts during both sequences, or they could change their probability along with the change in sequence structure. For example, if the sequences were 3-R-1-R-4-R-2-R and 3-R-2-R-1-R-4-R, respectively, stimulus on the 4^th^ location could be anticipated whenever a stimulus on the 1^st^ location was encountered two trials earlier (1-1-**4**, 1-2-**4**, 1-3-**4** and 1-4-**4** are all high frequency triplets during both phases of the study). We can refer to such stimuli as „high-high” (HH), indicating their probability in the two subsequent phases of the study (Fig. S2a). There are also stimuli that are less probable during both phases, for example encountering a stimulus on the 3^rd^ location after encountering a stimulus on the 1^st^ location two trials before (i.e. 1-1-**3**, 1-2-**3**, 1-3-**3** or 1-4-**3**). This can only happen on random trials, with an overall probability of 12.5%. These trials can be categorized as „low-low” (LL). Finally, there are cases when the probability of a stimulus changes with the change in the sequence structure. For example, encountering a stimulus on the 1^st^ location is highly probable during the first sequence when a participant encountered a stimulus on the 3^rd^ location two trials before (i.e. 3-1-**1**, 3-2-**1**, 3-3-**1** or 3-4-**1**); however, the same stimulus is less probable during the second sequence (encountering a stimulus on the 2^nd^ location would be probable in this case: 3-1-**2**, 3-2-**2**, 3-3-**2** or 3-4-**2**). If a stimulus is the probable continuation of its context during the first sequence, but less probable during the second, we can categorize it as „high-low” (HL); if it is less probable during the first sequence and probable during the second, we can categorize it as „low-high” (LH). Thus, the changes introduced between the Learning and the Rewiring Phase was based on 2^nd^ order dependencies in the ASRT sequence that was captured by these four triplet types. Each pair of sequences had the same amount of shared transitions (i.e. the proportion of HH, HL, LH and LL triplets was constant across participants).


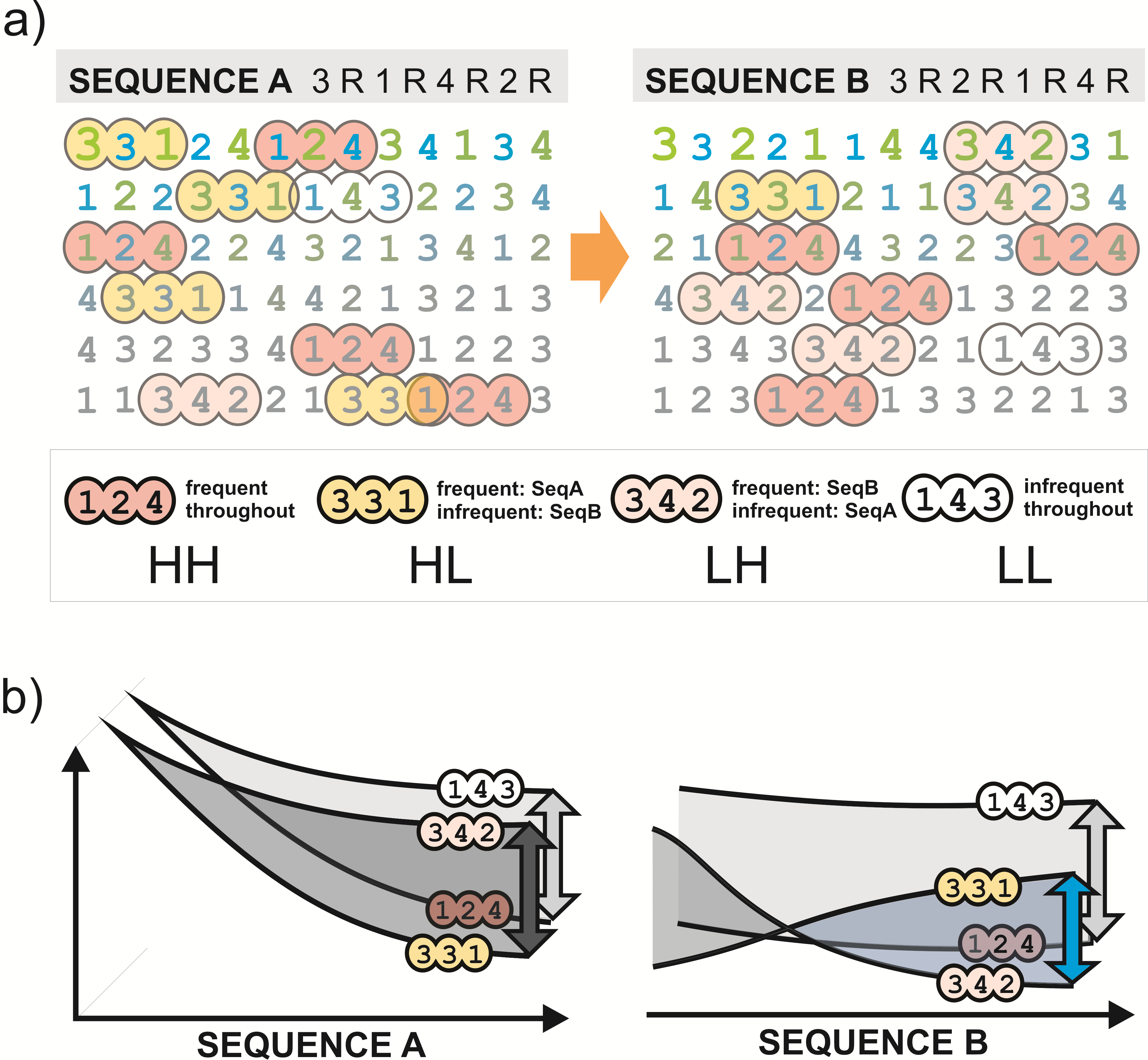


**Figure S2. Stimulus types as a function of shared vs. not shared transitional probabilities in Sequence A and Sequence B. (a)** Some of the triplets are frequent in both sequences – as they are „high frequency” during both Phases, we called them HH („high-high”) triplets (e.g. 1-2-4). Other transitions are frequent only in one of the sequences; the ones that are of high frequency in Sequence A but of low frequency in Sequence B (e.g. 3-3-1) are called HL triplets („high-low”); the ones with the opposite pattern (e.g. 3-4-2) are called LH triplets („low-high”). Finally, some of the triplets are of low probability during both sequences (e.g. 1-4-3) – these are called LL („low-low”) triplets. (**b)** Reaction times (RTs) to the final elements of the high frequency triplets are expected to be faster than RTs to the final elements of the low frequency triplets. Statistical Learning Effect (SLE) is the difference between RTs given to the two types of events (more probable vs. less probable) in both sequences. As particular transitions changed their frequency of occurrence when moving to Sequence B from Sequence A, different SLE-s were calculated. The SLE for the never changed transitional probabilities (HH vs. LL, e.g. 1-2-4 vs. 1-4-3) is shown by light grey arrows. SLE for the changed sequence parts is shown by two different colors; the dark grey arrow represents SLE before the swap in frequencies took place (3-3-1 vs. 3-4-2), while the blue arrow represents SLE after the change occurred (3-4-2 vs. 3-3-1). For the sake of clarity, we depicted these arrows at the end of learning, although average differences during the whole learning process were calculated.

## 1.3 Sequence combinations used in the current study

Different combinations of sequences were used in the experiment. Only 24 of the 30 possible combinations result in all four types of triplets (HH, LL, HL and LH) – or 12 out of 15 if we do not take order into account. The remaining sequence combinations would result in only LL, HL and LH triplets, no HH triplets (for example the sequences 1-R-2-R-3-R-4-R and 1-R-4-R-3-R-2-R have no high frequency triplets in common). All adequate combinations were used in the experiment (see Table S1) in a counterbalanced order. Importantly, we treated sequences 1-R-4-R-2-R-3-R-, 4-R-2-R-3-R-1-R-, 3-R-1-R-4-R-2-R- and 2-R-3-R-1-R-4-R- as being identical since they consist of the same triplets (just beginning at a different point of the sequence).

Table S1. Sequence combinations used in the experiment.

| **Sequence combinations** | **HH** | **LH or HL (depending on the order)** | **LL** |
| --- | --- | --- | --- |
| **1-R-2-R-3-R-4-R and 1-R-2-R-4-R-3-R** | 1-X-2  -  -  - | -  2-X-3 / 2-X-4  3-X-4 / 3-X-1  4-X-1 / 4-X-3 | 1-X-1, 1-X-3, 1-X-4,  2-X-1, 2-X-2,  3-X-2, 3-X-3,  4-X-2, 4-X-4 |
| **1-R-3-R-2-R-4-R and 1-R-3-R-4-R-2-R** | 1-X-3  -  -  - | -  2-X-4 / 2-X-1  3-X-2 / 3-X-4  4-X-1 / 4-X-2 | 1-X-1, 1-X-2, 1-X-4,  2-X-2, 2-X-3,  3-X-1, 3-X-3,  4-X-3, 4-X-4 |
| **1-R-4-R-2-R-3-R and 1-R-4-R-3-R-2-R** | 1-X-4  -  -  - | -  2-X-3 / 2-X-1  3-X-1 / 3-X-2  4-X-2 / 4-X-3 | 1-X-1, 1-X-2, 1-X-3,  2-X-2, 2-X-4,  3-X-3, 3-X-4,  4-X-1, 4-X-4 |
| **1-R-3-R-4-R-2-R and 1-R-4-R-3-R-2-R** | -  2-X-1  -  - | 1-X-3 / 1-X-4  -  3-X-4 / 3-X-2  4-X-2 / 4-X-3 | 1-X-1, 1-X-2,  2-X-2, 2-X-3, 2-X-4,  3-X-1, 3-X-3,  4-X-1, 4-X-4 |
| **1-R-2-R-3-R-4-R and 1-R-4-R-2-R-3-R** | -  2-X-3  -  - | 1-X-2 / 1-X-4  -  3-X-4 / 3-X-1  4-X-1 / 4-X-2 | 1-X-1, 1-X-3,  2-X-1, 2-X-2, 2-X-4,  3-X-2, 3-X-3,  4-X-3, 4-X-4 |
| **1-R-2-R-4-R-3-R and 1-R-3-R-2-R-4-R** | -  2-X-4  -  - | 1-X-2 / 1-X-3  -  3-X-1 / 3-X-2  4-X-3 / 4-X-1 | 1-X-1, 1-X-4,  2-X-1, 2-X-2, 2-X-3,  3-X-3, 3-X-4,  4-X-2, 4-X-4 |
| **1-R-2-R-4-R-3-R and 1-R-4-R-2-R-3-R** | -  -  3-X-1  - | 1-X-2 / 1-X-4  2-X-4 / 2-X-3  -  4-X-3 / 4-X-2 | 1-X-1, 1-X-3,  2-X-1, 2-X-2,  3-X-2, 3-X-3, 3-X-4,  4-X-1, 4-X-4 |
| **1-R-3-R-2-R-4-R and 1-R-4-R-3-R-2-R** | -  -  3-X-2  - | 1-X-3 / 1-X-4  2-X-4 / 2-X-1  -  4-X-1 / 4-X-3 | 1-X-1, 1-X-2,  2-X-2, 2-X-3,  3-X-1, 3-X-3, 3-X-4,  4-X-2, 4-X-4 |
| **1-R-2-R-3-R-4-R and 1-R-3-R-4-R-2-R** | -  -  3-X-4  - | 1-X-2 / 1-X-3  2-X-3 / 2-X-1  -  4-X-1 / 4-X-2 | 1-X-1, 1-X-4,  2-X-2, 2-X-4,  3-X-1, 3-X-3, 3-X-3,  4-X-3, 4-X-4 |
| **1-R-2-R-3-R-4-R and 1-R-3-R-2-R-4-R** | -  -  -  4-X-1 | 1-X-2 / 1-X-3  2-X-3 / 2-X-4  3-X-4 / 3-X-2  - | 1-X-1, 1-X-4,  2-X-1, 2-X-2,  3-X-1, 3-X-3,  4-X-2, 4-X-3, 4-X-4 |
| **1-R-3-R-4-R-2-R and 1-R-4-R-2-R-3-R** | -  -  -  4-X-2 | 1-X-3 / 1-X-4  2-X-1 / 2-X-3  3-X-4 / 3-X-1  - | 1-X-1, 1-X-2,  2-X-2, 2-X-4,  3-X-2, 3-X-3,  4-X-1, 4-X-3, 4-X-4 |
| **1-R-2-R-4-R-3-R and 1-R-4-R-3-R-2-R** | -  -  -  4-X-3 | 1-X-2 / 1-X-4  2-X-4 / 2-X-1  3-X-1 / 3-X-2  - | 1-X-1, 1-X-3,  2-X-2, 2-X-3,  3-X-3, 3-X-4,  4-X-1, 4-X-2, 4-X-4 |

The first pattern trial in each block was chosen randomly, so for example if the ASRT sequence was 3-R-1-R-4-R-2, then some of the blocks started as 3-R-1-R-4-R-2-R-3-R-1-R-4-R-2-R- while others started as 1-R-4-R-2-R-3-R-1-R-4-2 or 4-R-3-R-1-R-4-R-2-R-3-R- or 2-R-3-R-1-R-4-R-2-R-3. Note that the pattern is repeated in the block (10 times in each), thus changing the starting point does not lead to a different sequence (just as 123412341234 is the same as 234123412341).

In each combination, there was a partial overlap between Sequence A and Sequence B. Twenty-five percent of the originally high frequency triplets in Sequence A remained high frequency in Sequence B as well, while the remaining 75% became low frequency triplets. For example, Sequence A was 1–R–3–R–4–R–2 and Sequence B was 1–R–4–R–3–R–2 (see Line 4 in the table above). Following this example, out of the 16 originally high frequency triplets (i.e., 2–X–1, 1–X–3, 3–X–4, 4–X–2; X indicates the middle element of the triplet; i.e., 2–1–1, 2–2–1, 2–3–1, 2–4–1), four remained unchanged (triplets 2–X–1; HH triplets) and 12 high frequency triplets became low frequency ones (HL triplets).

Beyond the 16 high frequency triplets, there were 48 low frequency triplets for a given sequence. Out of these 48 low frequency triplets, 12 became high frequency (in the above example: 1–X–4, 4–X–3, 3–X–2; LH triplets), and 36 remained low frequency (e.g., 2–X–3, 2–X–4, 1–X–2, 4–X–1; LL triplets). In Table S1 we included which triplets corresponded to the categories of HH, HL, LH, and LL for each sequence pair combinations.

## 1.4 Calculation of Statistical Learning Effect (SLE)

In accordance with the original way of analysis[^1^](#_ENREF_1), RTs given to repetitions (e.g. 1-1-1) or trills (e.g. 1-3-1) were excluded, along with RTs of inaccurate responses and preparatory trials. Unlike the conventional analysis, we also excluded RTs given to pattern trials (except for the probe blocks, see Fig. 1 in the main text). If pattern trials were included, average RTs would have been lower for individuals performing the Explicit variant of the task, as they could explicitly anticipate stimuli on pattern trials (50% of all trials). Our aim was not to compare RTs when individuals knew in advance what the next stimulus was going to be versus when they had no explicit knowledge about this; we wanted to measure knowledge about the statistical structure which accompanies the alternating nature of random and pattern trials. Thus, by excluding pattern trials from analysis, we aimed to compare participants’ statistical knowledge under similar conditions (when they could not explicitly anticipate the stimuli, irrespective of the type of ASRT) (see “pure statistical learning” in Nemeth et al.[^2^](#_ENREF_2)).

We calculated median RTs given to random trials for each participant in each epoch for the four possible triplet types: HH, LL, HL and LH. Statistical Learning Effect (SLE) is the RT difference of responding to high frequency (probable) versus low frequency (less probable) trials. To get a positive value, we subtracted RTs given to high frequency trials from RTs given to low frequency trials that are usually slower. As we were specifically interested in the rewiring of learned sequences, we calculated 2 different SLE-s: one for those transitions (triplets) that did not change their frequency in the Rewiring Phase (SLE_NO REWIRING_ = RT_LL_ - RT_HH_ in both phases), and one for those that changed their frequency in the Rewiring Phase (SLE_REWIRING_ = RT_LH_ - RT_HL_ in the Learning Phase, and SLE_REWIRING_ = RT_HL_ - RT_LH_ in the Rewiring Phase), so that we obtained a positive value whenever participants showed learning of the currently valid statistical structure (Fig. S2b). The higher the SLE, the bigger the difference between RTs given to the more probable stimuli in contrast to the less probable stimuli under the current circumstances. In the Learning Phase, theoretically, there was no reason for SLEs to differ in magnitude as a function of later rewiring (SLE_NO REWIRING_ vs. SLE_REWIRING_). However, in the Rewiring Phase the two types of SLEs may differ if participants experience difficulties modifying their skill.

## 1.5 Calculation of anticipatory errors

To test whether participants learned to anticipate the most probable endings of triplets we looked at erroneous responses and classified each error either as being *nonanticipatory* (resulting in a low frequency triplet), *anticipation of sequence A* (when the erroneous key press completed a triplet that was frequent during the Learning Phase, although the stimulus was on another location), *anticipation of sequence B* (when the erroneous key press completed a triplet that was frequent during the Rewiring Phase, although the stimulus was on another location) or *anticipation of both sequences* (in the rare case when the resulting triplet is high frequency during both phases). As before, we only analysed those errors that were given to random elements intervening the pattern elements (leaving out the initial preparatory random elements in each block); and we excluded those errors that were given on trills or repetitions. If all the errors were independent of learning (i.e. they occurred randomly), then by chance 16.67% of them were expected to be anticipatory of sequence A; another 16.67% of them were expected to be anticipatory of sequence B; 5.56 % were expected to be anticipatory of both Sequence A and Sequence B; and the remaining 61.11% were expected to be nonanticipatory. A different error proportion was expected in the case of the probe epochs: owing to inclusion of the pattern elements (which always corresponded to high frequency triplets of the current sequence), chance levels for anticipations of the alternative sequence grew substantially. Specifically, the chance level for anticipatory errors of Sequence A in the Learning Phase was only 7.41%, while the chance level for anticipations of the other sequence (Sequence B) was 21.42% - and the reversed pattern hold for the Rewiring Phase. Our critical measure was whether anticipatory errors were more numerous than expected by chance, and whether the proportion of anticipations of Sequence A and Sequence B corresponded to the expected pattern - so in our analysis we only included *anticipatory errors of Sequence A* and *anticipatory errors of Sequence B* (leaving out anticipatory errors of both sequences and nonanticipatory errors). It must be noted that both kinds of anticipations may be above chance level, but these measures are related (to each other and to the other two kinds of errors). If one kind of errors is more numerous, it lowers the proportion of other kinds. Also, this measure does not tell anything about the total number of errors. Participants without errors on some epochs are excluded from this analysis due its within subject nature, as error proportions cannot be calculated for these epochs.

## 1.6 Tests for assessing the explicit knowledge about the sequence structures

### 1.6.1 Free Generation Task

According to the process dissociation framework[^3^](#_ENREF_3), intentional and automatic, nonintentional forms of memory can be separated by asking participants to a) intentionally include the learned material in their responses – the *inclusion condition*, and b) to intentionally exclude the previously learned material from their responses – the *exclusion condition*. If participants nevertheless include the learned material in the exclusion condition, knowledge of this material should be considered implicit; while performance in the inclusion condition is affected by both implicit and explicit knowledge. Comparing the performance under the two conditions can give us an estimate about the explicitness of the learning. Destrebecqz & Cleeremans[^4^](#_ENREF_4) proposed a ‘free generation’ task as a form of the process dissociation procedure specifically adapted to sequence learning paradigms. In the free generation task, stimuli appeared on the screen as a result of the corresponding buttonpresses, not the other way around. Thus, participants *generated the sequence,* both under inclusion and exclusion conditions.

As participants learned two sequences during the Learning and Rewiring Phase, they performed the free generation task twice on the third day, after the completion of the ASRT task: once for Sequence A and once for Sequence B, in random order. The stimuli that appeared in the free generation task looked exactly like those seen during the Learning and Rewiring Phases and mimicked the original task conditions: if a sequence was learned explicitly, stimuli appearing in the free generation task also alternated between the two colors. If a sequence was learned implicitly, stimuli appearing in the free generation task were always presented in the same color.

In our *free generation task* both the inclusion and exclusion conditions consisted of 4 blocks of 27 trials (that is, 25 triplets) each. Between the blocks, participants could pause for a few seconds if they needed. This way we obtained 100 generated triplets for both the inclusion and the exclusion conditions; the percentage of high frequency triplets could be easily calculated simply by counting the triplets that were high frequency transitions during learning. The question was whether participants differed in the explicitness they showed by this procedure as a function of the learning conditions of the particular sequence (explicit or implicit).

Even if a sequence remained entirely implicit, participants could have some general knowledge about it, for instance, that runs of three identical stimuli (e.g., 111, 222) were rare or runs of four identical stimuli (e.g., 1111, 2222) never occurred, etc. Performance in the free generation task could be affected by this knowledge. For example, if a participant pressed the same button in many consecutive trials, s/he could be sure that the resulting sequence was fundamentally different from the learned sequence. Such strategies result in data that could not be interpreted as reflecting explicit knowledge about the statistical structure of sequence (i.e., high vs. low frequency triplets), thus we excluded participants who pressed the same button in at least 50% of the free generation trials, and those who did not press at least one of the response buttons at all during the free generation trials (although these were somewhat arbitrary exclusion criteria; for example, when participants pressed the same button 49% of the trials had to be included by these criteria, even if we know that in the ASRT task all four stimuli occur equally often – 25% of trials). Our algorithm sure does not eliminate *all* strategies that may confound the measurement of implicit and explicit knowledge in the task, but at least eliminates those that most robustly affected the resulting sequences. This way 24 Implicit-Implicit, 19 Implicit-Explicit, and 18 Explicit-Explicit participants remained in the analysis.

### 1.6.2 Triplet Sorting Task

In our main analysis we were interested in participants’ ability to learn the statistical structure resulting from an ASRT sequence (some triplets being frequent, other triplets being infrequent). Thus, we used a triplet sorting task[^5^](#_ENREF_5) in which we presented all the 64 possible triplets (4x4x4) to participants – in each case, runs of three consecutive trials appeared on the screen, all initiated by the computer one after the other. Stimuli were identical in location and size to those seen during sequence learning – but the color of stimuli were always grey (independently of sequence learning conditions). When the presentation of the triplet was over, we asked participants to categorize that triplet either as a high frequency triplet or a low frequency triplet. As our participants actually learned two sequences (Sequence A and Sequence B), participants completed two triplet sorting tasks on the third day after the completion of the ASRT task: one for Sequence A and one for Sequence B, in random order.

## 1.7 Statistical analysis

Results were obtained using Mixed Design ANOVAs and Bonferroni-corrected post hoc tests if the omnibus ANOVA showed significant main effects or interactions. Sphericity was assessed with Mauchly’s Test, and if this precondition was not met, degrees of freedom were adjusted with the Greenhouse-Geisser method. Partial eta squared effect sizes are reported for significant main effects and interactions in ANOVA. Cohen’s d is reported for post hoc pairwise comparisons.

# 2. SUPPLEMENTARY RESULTS

##

## 2.1 Dynamics of the rewiring process in the experimental epochs compared across the Learning and Rewiring Phase

### 2.1.1 Statistical Learning Effect (SLE)

To analyse learning and rewiring on the first two days of the Experiment (see Fig. S3a), a 2 x 8 x 2 x 3 Mixed Design ANOVA was conducted on SLE-s with **PHASE** (Learning Phase or Rewiring Phase), **EPOCH** (1-8), **REWIRING** (change or no change in the frequency of particular transitions; SLE_NO REWIRING_ vs. SLE_REWIRING_) as within subject factors, and **GROUP** (Implicit-Implicit, Implicit-Explicit, Explicit-Explicit) as a between subject factor.

There was a significant main effect of **EPOCH**, *F*(5.584, 446.718) = 6.554, *MSE* =1073.133, *p* < .001, *η_p_^2^* = .076, as SLE-s increased as learning progressed in the two Phases. The significant main effect of **REWIRING**, *F*(1, 80) = 9.604, *MSE* = 1649.252, *p* = .003, *η_p_^2^* = .107, showed that on average SLE-s of the changed part of the sequence were lower than SLEs for the unchanged part of the sequence (*d* = .446). The **PHASE x GROUP** interaction also reached significance, *F*(2, 80) = 3.353, *MSE* = 1793.348, *p* = .040, *η_p_^2^* = .077. Post hoc tests showed that the average SLEs were significantly lower in the Rewiring Phase than in the Learning Phase in the case of the Implicit-Implicit group (*p* = .031, *d* = .575), and there was a trend toward the same effect in the Explicit-Explicit group (*p* = .091, *d* = .457) but not in the Implicit-Explicit group (*p* = .241, *d* = .309, the difference being in the other direction). Also, average SLEs of the Rewiring Phase in the Implicit-Implicit group were significantly lower than the same in the Implicit-Explicit group (*p* = .022, *d* = .757). No other paired comparisons reaching statistical significance (all *p*s > .373, all *d*s < .433).

There was a significant interaction of **PHASE x REWIRING**, *F*(1, 80) = 19.604, *MSE* = 1283.176, *p* < .001, *η_p_^2^* = .197. Post hoc tests showed that there was no difference between average SLEs of the (later) changed transitions and the unchanged transitions of the sequence in the Learning Phase (*p* = .568, *d* = .080). In the Rewiring Phase, on the other hand, the SLEs for the changed transitions were significantly lower (*p* < .001, *d* = .829). Also, SLEs for the changed part of the sequence was on average lower in the Rewiring Phase than in the Learning Phase (*p* < .001, *d* = .729), that is, rewiring was not as effective as the original learning, while performance of the unchanged transitions was comparable in the two phases (*p* = .151, *d* = .021).

Most importantly, there was a significant **PHASE** x **REWIRING** x **GROUP** interaction, *F*(2, 80) = 4.951, *MSE* = 1283.176, *p* = .009, *η_p_^2^* = .110, showing that the previously described difficulty in rewiring the original skill was not homogenous in the three groups. Post hoc tests revealed that SLEs for the rewired sequence part in the Rewiring Phase were smallest in the Implicit-Implicit group, being significantly lower than in the Explicit-Explicit (*p* = .028, *d* = .743) or Implicit-Explicit groups (*p* < .001, *d* = 1.198). The disadvantage of the rewired sequence part in the Rewiring Phase (in contrast to the unchanged sequence part in the same Phase) were apparent in the Implicit-Implicit (*p* < .001, *d* = 1.425) and the Explicit-Explicit groups (*p* = .008, *d* = .737), but not in the Implicit-Explicit group (*p* = .128, *d* = .406). Finally, the rewired SLEs in the Rewiring Phase were smaller than the original learning of these transitions in the Learning Phase for both the Implicit-Implicit (*p* < .001, *d* = 1.562) and Explicit-Explicit (*p* = .028, *d* = .850) groups, but again, such difficulty in rewiring was not apparent in the Implicit-Explicit group (*p* = .561, *d* = .155). There was no other significant main effect or interaction (all *p*s > .117, all *η_p_^2^* < .032).

In summary, the Implicit-Implicit group had more difficulty in rewiring the original skill than the other groups – thus rewiring of such skills may benefit from explicit knowledge about the sequence’s structure. As shown by 95% confidence intervals (see the blue line on Fig. S3a) rewiring started later in the Implicit-Implicit group, and was not statistically significant in the first half of the Rewiring Phase. In contrast, rewiring was evident as early as the first epoch of the Rewiring Phase in the case of the Implicit-Explicit and Explicit-Explicit groups.


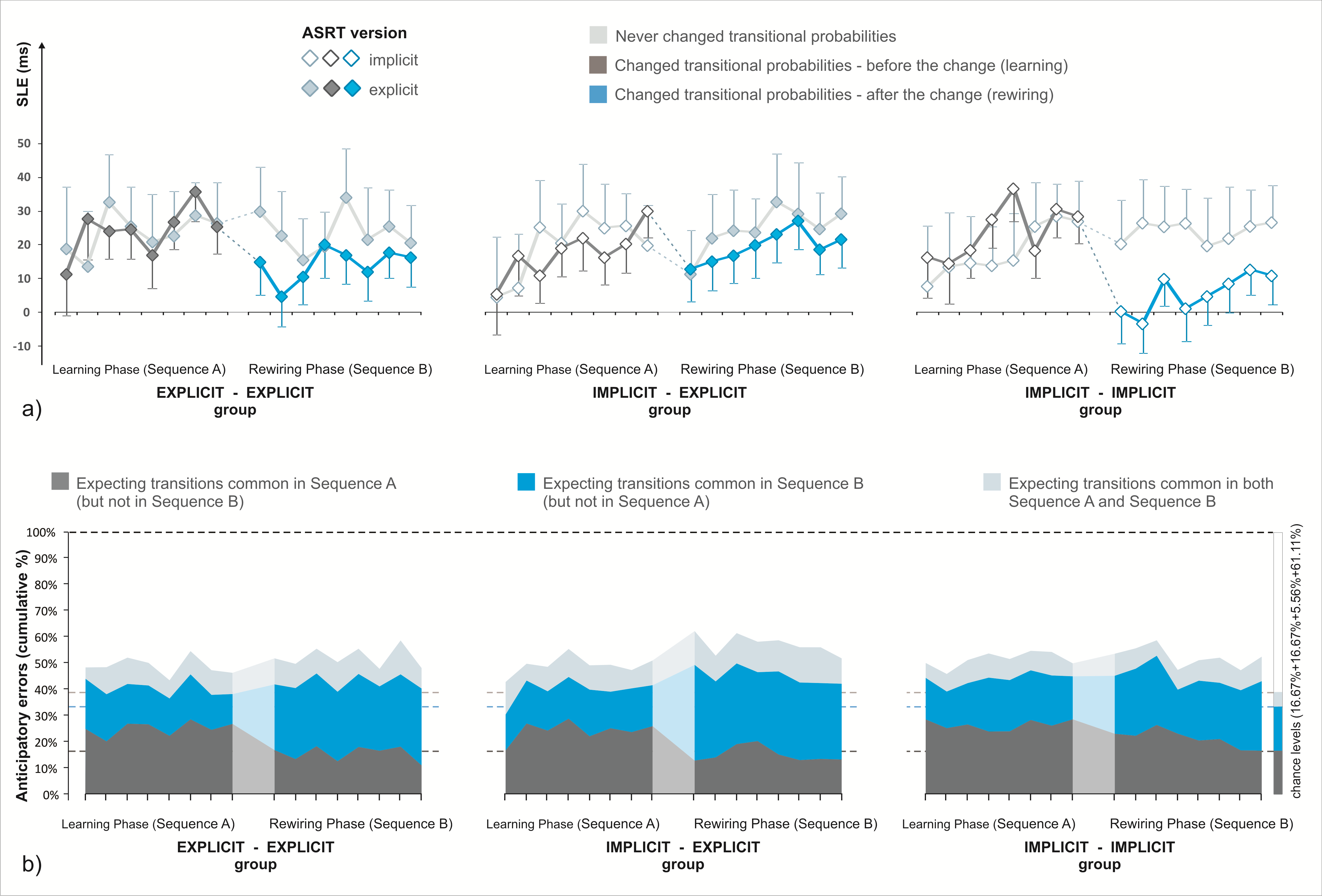


**Figure S3. Learning and rewiring – detailed graphs**. **(a)** The magnitude of Statistical Learning Effect (SLE) indicates the difference of RTs given to frequent transitions (probable stimuli) in contrast to rare transitions (less probable stimuli). Some of the transitions had constant frequency in the Learning Phase and Rewiring Phase (unchanged transitions, dark grey line), while other transitions swapped their frequency – previously rare transitions became frequent in the Rewiring Phase, and vice versa (changed transitions). Adapting to the changed statistical structure in the Rewiring Phase was shown to be more difficult than learning the contingencies in the first place in the Learning Phase. This was shown by SLEs being – on average – lower for the changed transitions after the change in frequencies took place in the Rewiring Phase (blue line) than before the change (dark grey line). This difficulty was most pronounced in the Implicit-Implicit group, and less pronounced in the Implicit-Explicit group. Error bars represent 95% confidence intervals. **(b)** When less probable stimuli came up, participants sometimes errorneously pressed the key corresponding to the most probable stimulus – these errors are called anticipatory errors. As two (partly) different sequences were taught, we differentiated between anticipations of Sequence A’s most probable stimuli (dark grey area), that of Sequence B’s most probable stimuli (blue area), and those that could be considered as anticipations of both (light grey area). Chance levels for anticipatory errors are shown by the dotted lines. We were mainly interested in anticipations that either corresponded to Sequence A or to Sequence B (dark grey and blue areas). Each group showed adaptation to the current sequence, as anticipations for Sequence A were above chance level in the Learning Phase, while anticipations of Sequence B were above chance level in the Rewiring Phase. The Implicit-Implicit group additionaly showed above chance level anticipations of Sequence A during the Rewiring Phase, indicating the continuing influence of their knowledge gained in the Learning Phase.

###

### 2.1.2 Anticipatory Errors

To look at anticipations of Sequences A and B during the Learning Phase and the Rewiring Phase (see Fig. S3b), a 2 x 8 x 2 x 3 Mixed Design ANOVA was conducted with **PHASE** (Learning Phase or Rewiring Phase), **EPOCH** (1-8) and **ANTICIPATION** (anticipation of Sequence A vs. anticipation of Sequence B) as within subject factors and **GROUP** (Implicit-Implicit, Implicit-Explicit and Explicit-Explicit) as a between subjects factor.

There were no significant main effects (all *p*s > .113, all *η_p_^2^* < .036). The interaction of **ANTICIPATION x GROUP** showed a trend towards significance, *F*(2, 71) = 2.544, *MSE* = 904.244, *p* = .086, *η_p_^2^* = .067. Post hoc tests revealed that, overall, anticipations of Sequence A and Sequence B did not differ in either group (all *p*s > .106, all *d*s < .548); however, there was a difference between the Implicit-Implicit and Implicit-Explicit groups when anticipating transitions common in Sequence A: the Implicit-Implicit group made more such anticipations then the Implicit-Explicit group (*p* = .050, *d* = .714). No other paired comparison reached significance, all *p*s > .163, all *d*s < .600).

The **PHASE x ANTICIPATION** interaction was significant, *F*(1, 71) = 86.707, *MSE* = 572.289, *p* < .001, *η_p_^2^* = .550. As expected (and as shown by post hoc comparisons), anticipations of Sequence A were more pronounced in the Learning Phase than anticipations of Sequence B, and vice versa in the Rewiring Phase (both *p* < .001, both *d* > 1.061). During the time course of learning, anticipations of Sequence A were more common in the Learning Phase than in the Rewiring Phase; while anticipations of Sequence B were more common in the Rewiring Phase than in the Learning Phase (both *p* < .001, both *d* > .979). Most importantly, the **PHASE x ANTICIPATION x GROUP** interaction was also significant, *F*(2, 71) = 3.917, *MSE* = 572.289, *p* = .024, *η_p_^2^* = .099. Post hoc tests revealed that the previously described pattern was observed in all experimental groups, although effect sizes were substantially smaller in the case of the Implicit-Implicit group (both *d* < 0.672) than in the other groups (all *d*s > 1.226). Looking at the Learning Phase alone, groups did not differ in terms of anticipations of Sequence A (all *p*s > .999, *d*s < .263) and Sequence B (all *p*s > .999, *d*s < .285). In all groups, anticipations of Sequence A outnumbered anticipations of Sequence B in this Phase (all *p*s < .001, all *d*s > 1.058). Looking at the Rewiring Phase alone, however, showed us a more complicated pattern. In this phase anticipations of Sequence B outnumbered anticipations of Sequence A only in the Explicit-Explicit and Implicit-Explicit groups (both *p* < .001, both *d* > .1.133) but not in the Implicit-Implicit group (*p* = .529, *d* = .225). Anticipations of Sequence B were significantly less common in the Implicit-Implicit group than in the Implicit-Explicit group (*p* = .047, *d* = .721), while anticipations of Sequence A were significantly more common in the Implicit-Implicit group than both in the Implicit-Explicit and the Explicit-Explicit groups (both *p* < .036, both *d* > .795). The latter findings may be interpreted as a shift toward anticipating Sequence A at the expense of anticipating Sequence B in the case of the Implicit-Implicit group.

## 2.2 Testing the efficiency of the rewiring process in the experimental epochs of the Follow-up Phase

On the third day of the experiment, participants performed the sequence that was learned in the Learning Phase (Sequence A) as well as the sequence that was learned in the Rewiring Phase (Sequence B). The order of these mini-epochs was counterbalanced across participants. We were interested in the consolidation of their knowledge about the statistical structure of the task, and particularly, whether they could re-adapt to the characteristics of the Learning Phase or not (that is, whether the statistical learning on the first day was overwritten by the statistical learning on the second day, or they existed in parallel). This analysis provides information about retroactive interference effects (in addition to proactive effects assessed earlier).

### 2.2.1 Statistical Learning Effect (SLE)

We conducted a 2 x 2 x 3 Mixed Design ANOVA on SLEs with **SEQUENCE** (the same conditional probabilities as in the Learning Phase - that is, Sequence A; or the same conditional probabilities as in the Rewiring Phase - that is, Sequence B) and **REWIRING** (change or no change in the frequency of particular transitions) as within subject factors, and **GROUP** (Implicit-Implicit, Implicit-Explicit, and Explicit-Explicit) as a between subject factor.

There was a significant main effect of **SEQUENCE,** *F*(1, 79) = 6.245, *MSE* = 891.475, *p* = .015, *η_p_^2^* = .073, as higher statistical knowledge was expressed for Sequence B (that is, for the conditional probabilities that corresponded to the Rewiring Phase on the second day of the study), than for Sequence A (*d* = .404). There was also a significant main effect of **REWIRING**, *F*(1, 79) = 9.574, *MSE* = 943.423, *p* = .003, *η_p_^2^* = .108, as higher statistical knowledge was expressed for those transitions that never changed their frequency of occurrence than for those that changed (*d* = .506). No other main effect or interaction reached significance, all *p*s > .284, *η_p_^2^*  < .031.

### 2.2.2 Anticipatory errors

There were two participants who made no errors when performing one of the Sequences - analysis was conducted on the remaining 27 (Explicit-Explicit), 28 (Explicit-Implicit) and 26 (Implicit-Implicit) participants. Chance level for both kinds of anticipations (anticipations of Sequence A and anticipations of Sequence B) was 16.67%. To assess anticipations on the third day of the study, a 2 x 2 x 3 Mixed Design ANOVA was conducted with **SEQUENCE** (Sequence A vs. Sequence B) and **ANTICIPATION** (anticipation of transitions common to Sequence A only vs. anticipation of transitions common to Sequence B only) as within subject factors and **GROUP** (Implicit-Implicit, Implicit-Explicit, and Explicit-Explicit) as a between subjects factor.

There was a trend towards a main effect of **GROUP**, *F*(2, 78) = 2.582, *MSE* = 292.754, *p* = .082, *η_p_^2^* = .062. However, post hoc pairwise comparisons revealed no significant differences between groups, all *p*s >.141, *d*s < .574. We also found a significant interaction of **SEQUENCE x ANTICIPATION**, *F*(1, 78) = 13.815, *MSE* = 596.817, *p* < .001, *η_p_^2^* = .150. Post hoc tests revealed that when performing Sequence A, anticipations of Sequence A were more common than anticipations of Sequence B (*p* = . 004, *d* = .533), and than what might have been expected by chance, *CI95%* [24.699, 34.391]. Anticipations of Sequence B did not differ from chance level, *CI95%* [15.318, 22.844]. When performing Sequence B, on the other hand, anticipations of Sequence B outnumbered anticipations of Sequence A (*p* = .009, *d* = .503), and were more numerous than expected by chance, *CI95%* [23.822, 32.291]. Anticipations of Sequence A did not differ from chance level, *CI95%* [14.024, 22.644]. From another point of view, anticipations of Sequence A were significantly more pronounced when performing Sequence A than when performing Sequence B, and vice versa (both *p* < .003, *d* > .494). This pattern of results indicate no proactive or retroactive interference, as participants were able to quickly adapt to the changed statistical conditions.

## 2.3 Dynamics of the rewiring process in the probe epochs compared across the Learning and Rewiring Phase

### 2.3.1 Statistical Learning Effect (SLE)

SLEs were calculated for the probe epochs of the Learning and Rewiring Phase. During these short epochs at the beginning, in the middle and at the end of each phase, trials were not cued, thus the task remained implicit for all participants. This manipulation made it possible to compare groups under uniform experimental conditions. Also, as there were no cued trials during these epochs, there was no need to exclude pattern trials. Correspondingly, although these epochs were substantially shorter than the experimental epochs, about the same number of trials were analysed. It must be noted, though, that the proportion of high frequency (more probable) and low frequency (less probable) combinations are different if the analysis includes pattern trials, as in this case there are more high frequency triplets than low frequency triplets, making the median RTs of the latter a bit noisier then the former.

Similarly as before, a 2 x 8 x 2 x 3 Mixed Design ANOVA was conducted on SLEs shown in Fig. S4a with **PHASE** (Learning Phase vs. Rewiring Phase), **PROBE** **EPOCH** (1-3) and **REWIRING** (change or no change in the frequency of particular transitions) as within subject factors, and **GROUP** (Implicit-Implicit, Implicit-Explicit, and Explicit-Explicit) as a between subject factor.

The main effect of **PROBE EPOCH** was significant, *F*(1.633, 130.680) = 34.447, *MSE* = 947.431, *p* < .001, *η_p_^2^* = .301, as statistical knowledge indicated by SLEs became higher as learning progressed. The main effect of **REWIRING** showed a trend towards significance, *F*(1, 80) = 2.870, *MSE* = 1157.559, *p* = .094, *η_p_^2^* = .035, as overall SLEs were higher for the never changed parts of the sequence than for the changed part (*d* = .224). We found a significant **PHASE x REWIRING** interaction, *F*(1, 80) = 18.905, *MSE* = 858.840, *p* < .001, *η_p_^2^* = .191. Post hoc tests revealed that although there was no difference between the later to be changed transitions and the unchanged transitions during the Learning Phase (*p* = .166, *d* = .202), there was a substantial advantage of the unchanged transitions during the Rewiring Phase (*p* < .001, *d* = .737). During the time course of learning, there was a significant improvement in the performance on the unchanged parts of the sequence from the Learning Phase to the Rewiring Phase (learning for these transitions continued in the Rewiring Phase, *p* = .001, *d* = .432); while there was a significant drop in statistical knowledge for the rewired sequence parts in the Rewiring Phase (compared to the original learning in the Learning Phase, *p* = .009, *d* = .414).

There was also a significant **PHASE x REWIRING x GROUP** interaction, *F*(2, 80) = 3.908, *MSE* = 858.840, *p* = .024, *η_p_^2^* = .089, meaning that the previously described pattern was not homogenous in the three groups. Post hoc comparisons revealed that the Implicit-Implicit group showed an advantage of the later to be rewired sequence parts during the Learning Phase (*p* = .013, *d* = .654), while no difference between the later changed and unchanged sequence parts were observed in the other two groups (both *p* > .809, both *d* < .063). The difficulty of rewiring was apparent in all three groups, as participants responded faster to the unchanged transitions than to the recently changed transitional probabilities in the Rewiring phase (Explicit-Explicit group: *p* = .060, *d* = .541; Implicit-Explicit group: *p* = .023, *d* = .645, Implicit-Implicit group: *p* < .001, *d* = 1.097). It could also be observed that learning of the unchanged transitional probabilities continued in the Rewiring Phase, as statistical knowledge for these transitions were higher in the Rewiring Phase than in the Learning Phase for all groups (although this pattern was significant only in the Implicit-Implicit group: *p* = .001, *d* = .767; a trend was observed for the Explicit-Explicit group: *p* = .083, *d* = .389, and nonsignificant in the Implicit-Explicit group: *p* = .405, *d* = .182). Statistical learning for the recently changed transitional probabilities, on the other hand, was smaller than the original learning before rewiring for all groups (although it only reached significance in the Implicit-Implicit group: *p* = .001, *d* = .913; all other *p* > .287, *d* < .296). In addition, there was an interaction of **PROBE EPOCH x REWIRING x GROUP**, *F*(4, 160) = 2.969, *MSE* = 649.224, *p* = .021, *η_p_^2^* = .069, indicating that the previously described effects varied as a function of probe epochs in the Learning and Rewiring Phases. A detailed graph depicting all levels of this interaction is shown in Fig. S4a.

In summary, analysis of the probe epochs strengthened our results observed in the experimental epochs. Rewiring of recently changed transitional probabilities was shown to be harder for the Implicit-Implicit group than for the other two groups who rewired with the help of explicit cues, although – by the end of the Rewiring Phase – all three groups showed adaptation to the new statistical structure (shown by 95% CIs on the blue lines on Fig. S4a).


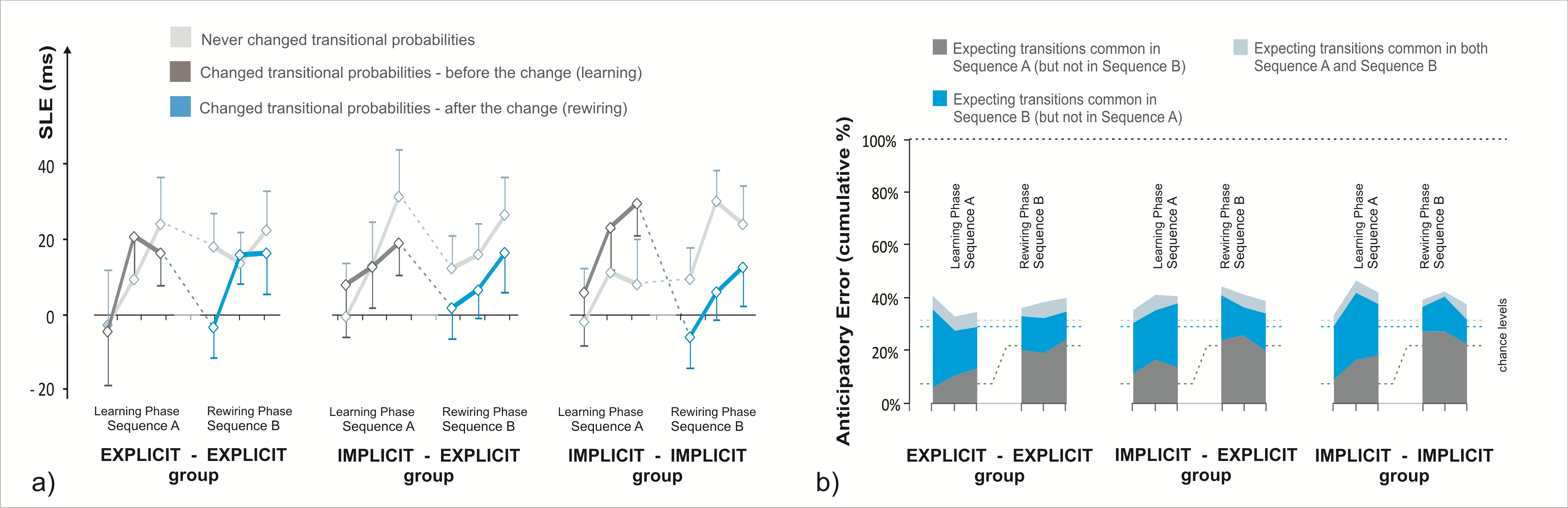


**Figure S4. Learning and Rewiring in the Probe epochs – detailed graphs**. **(a)** Adapting to the changed statistical structure in the Rewiring Phase was shown to be more difficult than learning the contingencies in the first place in the Learning Phase. This was shown by SLEs being – on average – lower for the changed transitions after the change in frequencies took place in the Rewiring Phase (blue line) than before the change (dark grey line). Error bars represent 95% CIs. **(b)** Anticipatory errors either corresponded to Sequence A or to Sequence B (dark grey and blue areas). Participants could adapt to both statistical regularities as anticipations of Sequence A were higher than expected by chance when performing Sequence A in the Learning Phase; similarly, anticipations of Sequence B were higher then expected by chance when performing Sequence B in the Rewiring Phase. Chance levels for anticipatory errors are shown by the dotted lines.

### 2.3.2 Anticipatory errors

Participants without errors on some probe epochs were excluded from the analysis due its within subject nature, as error-proportions could not be calculated for these probe epochs. Analysis was performed on the remaining 23 (Explicit-Explicit), 26 (Implicit-Explicit) and 23 (Implicit-Implicit) participants. We conducted a 2 x 3 x 2 x 3 Mixed Design ANOVA on the anticipatory data shown in Fig. S4b with **PHASE** (Learning Phase vs. Rewiring Phase), **PROBE** **EPOCH** (1-3) and **ANTICIPATION** (anticipation of Sequence A vs. anticipation of Sequence B) as within subject factors and **GROUP** (Implicit-Implicit, Implicit-Explicit, and Explicit-Explicit) as a between subject factor.

The ANOVA revealed only one significant interaction: a **PHASE x ANTICIPATION** interaction, *F*(1, 69) = 66.157, *MSE* = 308.436, *p* < .001, *η_p_^2^* = .489. Post hoc tests revealed that – as expected even by chance levels – there were significantly more anticipations of Sequence B than anticipations of Sequence A during the Learning Phase; and vice versa (both *p* < .001, *d* > .765); furthermore, 95% confidence intervals indicated that in the Learning Phase the anticipations of Sequence A were more pronounced than expected by chance, *CI95%* [11.139, 14.752], while anticipations of Sequence B did not differ from what we might have expected by chance, *CI95%* [18.091, 24.229]. In the Rewiring Phase, anticipatory errors of Sequence A did not differ from what we might have expected by chance, *CI95%* [20.760, 26.173]; while anticipations of Sequence B were more numerous than expected by chance, *CI95%* [9.894, 14.525]. Thus, anticipatory errors indicated that participants indeed learned to anticipate the most probable continuation of the previous trials both before and after rewiring the transitional probabilities. Post hoc tests also indicated that anticipations of Sequence A were of greater proportion during the Rewiring Phase than during the Learning Phase, and vice versa for anticipations of Sequence B (both *p* < .001, *d* > .773) – this pattern was expected even by chance, thus not providing additional information to our evaluation of the results. Critically, the interaction of **PHASE x ANTICIPATION x GROUP** was not significant, *F*(2, 69) = 0.580, *MSE* = 308.436, *p* = .563, *η_p_^2^* = .017, suggesting that the previously described pattern was similar across the three experimental groups. This result did not rule out the possibility that differences existed, though, as the pattern can be very similar across groups even if some anticipations are above or below chance levels for some of the groups – this being the most important information we tried to uncover. Finally, there was a trend towards a **PROBE EPOCH x ANTICIPATION x GROUP** interaction, *F*(4, 138) = 2.013, *MSE* = 300.555, *p* = .096, *η_p_^2^* = .055. A detailed graph depicting all levels of the interaction is shown in Fig. S4b.

## 2.4 Testing the efficiency of the rewiring process in the probe epochs of the Follow-up Phase

### 2.4.1 Statistical Learning Effect (SLE)

SLEs were calculated for probe epochs of the Follow-up Phase as previously described for the Learning and Rewiring Phase. We conducted a 2 x 2 x 3 Mixed Design ANOVA on SLEs shown in Fig. S5a with **SEQUENCE** (Sequence A vs. Sequence B) and **REWIRING** (change or no change in the frequency of particular transitions) as within subject factors, and **GROUP** (Implicit-Implicit, Implicit-Explicit, and Explicit-Explicit) as a between subject factor.

There was a significant main effect of **REWIRING**, *F*(1, 80) = 26.449, *MSE* = 585.105, *p* < .001, *η_p_^2^* = .248, as higher statistical knowledge was expressed for those transitions that never changed their transitional probability than for those that changed (*d* = .852). No other main effect or interaction reached significance, all *p*s > .313, *η_p_^2^*  < .030. This pattern of results indicates only proactive but no retroactive interference.

**
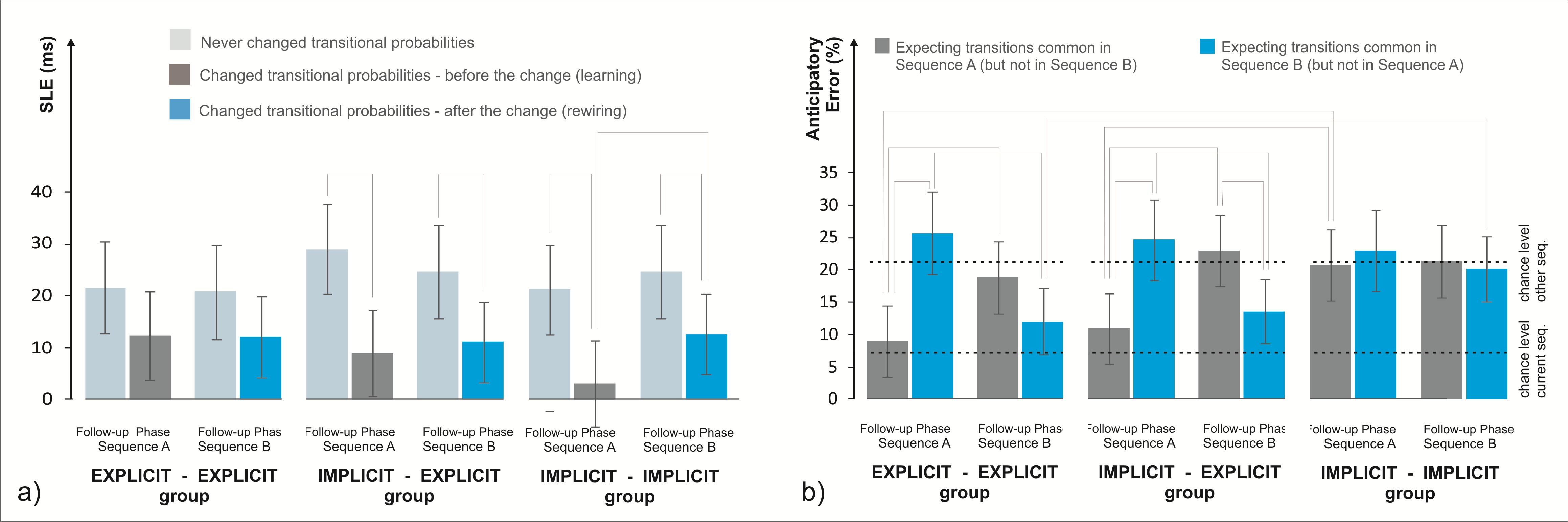
**

**Figure S5. Consolidation of learning in the Probe epochs of the Follow-up Phase**. **(a)** The magnitude of SLE indicates the difference of RTs given to frequent transitions (probable stimuli) in contrast to rare transitions (less probable stimuli). Some of the transitions had constant frequency in both Sequences (unchanged transitions, dark grey line), while other transitions were frequent in only one of the Sequences, not in the other (changed transitions; dark grey bars for Sequence A and blue bars for Sequence B). Performance was better on the unchanged transitions than on changing transitions in all groups. No group differences were observed. **(b)** Anticipatory errors of Sequence A’s most probable stimuli are shown in dark grey bars, and that of Sequence B’s most probable stimuli are shown in blue bars. Chance levels for anticipatory errors are shown by the dotted lines (there is a lower chance level of anticipatory errors of Sequence A when performing Sequence A than when performing Sequence B, and vice versa). Participants could readapt to both statistical regularities as anticipations of Sequence A were higher than expected by chance when performing Sequence A in the Follow-up Phase; similarly, anticipations of Sequence B were higher than expected by chance when performing Sequence B in the Follow-up Phase. Error bars represent 95% CIs.

### 2.4.2 Anticipatory errors

Anticipatory errors for the probe epochs of the Follow-up Phase were calculated as previously described for the Learning and Rewiring Phase. A 2 x 2 x 3 Mixed Design ANOVA on the anticipatory errors shown in Fig. S5b was conducted with **SEQUENCE** (Sequence A vs. Sequence B) and **ANTICIPATION** (anticipation of transitions common to Sequence A only vs. anticipation of transitions common to Sequence B only) as within subject factors and **GROUP** (Implicit-Implicit, Implicit-Explicit, and Explicit-Explicit) as a between subjects factor.

The ANOVA revealed a significant main effect of **GROUP**, *F*(2, 79) = 3.566, *MSE* = 193.010, *p* = .033, *η_p_^2^* = .083. Post hoc tests showed that this was caused by the Implicit-Implicit group making – on a trend level - more anticipations of Sequence A and B (overall) than the Explicit-Explicit group (*p* = .070, *d* = .705). No other paired comparison reached statistical significance, both *p* > .434, *d* < .444. We also found a significant **SEQUENCE x ANTICIPATION** interaction, *F*(1, 79) = 17.558, *MSE* = 324.940, *p* < .001, *η_p_^2^* = .182. Post hoc tests revealed a pattern consistent with chance levels, that is, higher levels of anticipations of Sequence B when performing Sequence A, and vice versa (both *p* < .011, *d* > .468); and higher levels of Sequence B during Sequence A than during Sequence B - and vice versa (both *p* < 0.03, *d* > .503). Solely on the basis of the ANOVA we could not infer anything about anticipatory errors; confidence intervals, on the other hand, provide some interesting details. Anticipations of Sequence A when performing Sequence A exceeded chance levels, *CI95%* [10.856, 17.527], and so did anticipations of Sequence B when performing Sequence B, *CI95%*[11.598, 18.029]. Anticipations of Sequence A, on the other hand, did not exceed chance levels when performing Sequence B, *CI95%*[18.067, 25.015], and vice versa, *CI95%*[20.679, 28.599].

Finally, there was a trend towards a **SEQUENCE x ANTICIPATIONS x GROUP** interaction, *F*(2, 79) = 2.777, *MSE* = 324.940, *p* = .068, *η_p_^2^* = .066, indicating that the previously described pattern of results was not the same in the three experimental groups. Post hoc tests revealed that the pattern expected by chance was apparent in the Explicit-Explicit and Implicit-Explicit groups (all *p*s < .039, all *d*s > .658), but not in the Implicit-Implicit group where anticipations of Sequence A and Sequence B were equally high both when performing Sequence A and when performing Sequence B (all *p*s > .551, *d*s < .213); additionally, 95% CIs indicated that the Implicit-Implicit group showed above-chance level of anticipations of Sequence A when performing Sequence A, *CI95%*[ 15.069, 27.189], and above-chance level of anticipations of Sequence B when performing Sequence B, *CI95%*[14.050, 25.736]. Based on confidence intervals, the Implicit-Explicit group also showed above-chance level anticipations of Sequence B when performing Sequence B, *CI95%* [8.377, 19.126].

In addition to these effects, there were differences between groups regarding the proportion of anticipations of Sequence A (when performing Sequence A); the Implicit-Implicit group showed significantly higher rates than the Explicit-Explicit group (*p* = .023, *d* = 836), and – on a trend level - higher rates than the Implicit-Explicit group (*p* = .080, *d* = .680). Also, there were differences between groups regarding the proportion of anticipations of Sequence B when performing Sequence B; the Implicit-Implicit group showed higher rates than the Explicit-Explicit group (on a trend level, *p* = .080, *d* = 688). No other paired comparison reached significance, all *p*s > .381, all *d*s < .465.

## 2.5 Testing the explicit knowledge acquired about the sequence structures

### 2.5.1 Free Generation Task

We conducted a Mixed Design ANOVA on the percentage of generated high frequency triplets with **SEQUENCE** (Sequence A vs. Sequence B) and **CONDITION** (Inclusion vs. Exclusion) as within subjects factors and **GROUP** (Implicit-Implicit, Implicit-Explicit, and Explicit-Explicit) as a between subject factor. To understand the results of such a factorial ANOVA, we have to keep in mind that the average explicitness of the task differed between the sequences and the conditions; the Explicit-Explicit group learned both sequences explicitly, thus the average explicitness was highest for this group; the Implicit-Explicit group learned Sequence A implicitly but Sequence B explicitly; while the Implicit-Implicit group learned both sequences implicitly (thus the average explicitness was lowest for this group). On the other hand, Sequence A was learned explicitly by only one of the groups (the Explicit-Explicit group), while Sequence B was learned explicitly by both the Implicit-Explicit and the Explicit-Explicit groups, making the average explicitness for Sequence B higher than that of Sequence A.

We found a significant main effect of **SEQUENCE**, *F*(1, 58) = 6.611, *MSE* = 122.210, *p* = .013, *η_p_^2^* = .102, as participants generated – on average – more high frequency triplets for Sequence B than for Sequence A (*d* = .502). There was also a significant main effect of **GROUP**, *F*(2, 58) = 24.295, *MSE* = 88.008, *p* < .001, *η_p_^2^* = .456, as the number of generated high frequency triplets was highest for the Explicit-Explicit group (*CI95%* [36.565, 40.991], significantly differing from the other groups, both *p* < .002, *d* > 1.327); and lowest for the Implicit-Implicit group (*CI95%* [26.667, 30.50], significantly differing from the other two groups, *p* < .014, *d* > .947).

More importantly, there was a significant **SEQUENCE x GROUP** interaction, *F*(2, 58) = 4.623, *MSE* = 122.210, *p* = .014, *η_p_^2^* = .138. Post hoc tests revealed that the interaction was caused by higher explicitness if the learning was explicit than when learning was implicit (see Fig. S6). That is, for Sequence A the Explicit-Explicit group outperformed the other two groups, both *p* < .002, *d* > 1.272 (the other groups did not differ from each other, *p* > .999, *d* = .137) – in accordance with this group being the only one to learn Sequence A explicitly. For Sequence B, on the other hand, a disadvantage of the Implicit-Implicit group was apparent – they generated significantly *less* high frequency triplets than the Implicit-Explicit and Explicit-Explicit groups, both *p* < .002, *d* > 1.262 (the latter two not differing from each other, *p* = .832, *d* = .379). The number of generated high frequency triplets for the two sequences did not differ from each other in the case of the Implicit-Implicit (*p* = .620, *d* = .160) and Explicit-Explicit (*p* = .263, *d* = .428) groups; but it was higher for Sequence B in the case of the Implicit-Explicit group (*p* = .001, *d* = 1.330) – as this was the only group where learning instructions differed for the two sequences. Thus, these results show that participants indeed gained more explicit knowledge about the regularities when they performed the explicit version of the task and were asked to keep track of these regularities.


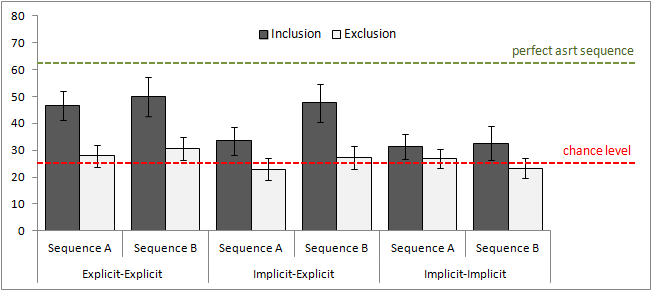


**Figure S6. Percentages of generating high frequency triplets in the free generation task.** Participants in the implicit conditions performed well below of those in the explicit conditions when they were asked to generate similar sequences as the ones that they encountered during the experiment.

We also found a significant main effect of **CONDITION**, *F*(1, 58) = 74.559, *MSE* = 151.926, *p* < .001, *η_p_^2^* = .562, as more high frequency triplets were generated under the inclusion condition than under the exclusion condition (*d* = 1.785). In addition, there was a significant **CONDITION x GROUP** interaction, *F*(2, 58) = 5.615, *MSE* = 853.092, *p* = .006, *η_p_^2^* = .162. Post hoc tests revealed that the previously described effect was only apparent under the inclusion condition (all *p*s < .028, all *d*s > .933), and the groups generated similar number of high frequency triplets under the exclusion condition (all *p*s > .163, all *d*s < .652). Overall, all groups generated more high frequency triplets under the inclusion condition than under the exclusion condition (all *p*s < .010, all *d*s > .900). When participants learned the sequences implicitly, this result of more high frequency triplets in the inclusion vs. exclusion conditions can be caused by a more general knowledge/belief about the task structure, not the awareness of the particular high vs. low frequency transitional probabilities per se (although we cannot totally rule this out).

Inclusion and exclusion strategies may differ for implicit and explicit learners, and consequently, the difference between inclusion and exclusion performance may mean different things in the two groups. For the explicit group, the instructions are quite straightforward as the alternating sequence was explicitly cued for them during the Learning/Rewiring Phases. Consequently, they could more easily generate the known alternating sequence (inclusion condition) and also could know what sequence to not generate (exclusion condition). For the implicit learners the instructions might have been more puzzling: they might have generated a seemingly random sequence just as they experienced throughout the task (inclusion condition) vs. a sequence intentionally so that it does not seem random (exclusion condition). These cases can lead to similar outcomes, but it does not necessarily mean explicit knowledge about the alternating sequence (actually it may indicate that the sequence was perceived absolutely random, and thus the generation under exclusion condition is something (anything) non-random. In line with this argument, Fu and colleagues[^6^](#_ENREF_6) showed that the difference between inclusion and exclusion scores could be based either on rules and memory (more explicit processes) but also on intuition (more implicit processes). Consequently, this task *alone* is not suited to measure the conscious status of structural knowledge[^7^](#_ENREF_7).

Importantly, significant group differences were found under the inclusion condition that reflect the effect of the explicit instructions. The results of the Triplet Sorting Task (see the next section) further supports the interpretation that knowledge of the underlying structure in the ASRT task remained implicit for participants in the implicit conditions.

### 2.5.2 Triplet Sorting Task

We were interested in how accurately participants sorted transitions (triplets) for Sequence A and Sequence B. We conducted a Mixed design ANOVA with **SEQUENCE** (Sequence A vs. Sequence B) as a within-subject factor and **GROUP** (Implicit-Implicit, Implicit-Explicit, and Explicit-Explicit) as a between subject factor. Due to technical errors, data for 7 participants were lost, all from the Implicit-Implicit group; therefore the ANOVA was conducted on the data from the remaining participants.

Overall accuracy was 50.208% (*SEM* = 0.987, *CI95%* [48.238%, 52.178%]), thus not significantly different from what we would expect by chance. There was a trend towards a main effect of **SEQUENCE**, *F*(1, 69) = 2.791, *MSE* = 42.823, *p* = .099, *η_p_^2^* = .039, participants being on average more accurate in the case of Sequence B than Sequence A (*d* = .191) – a fact possibly reflecting that knowledge of Sequence A in part became overwritten by the knowledge of Sequence B. In spite of the trend towards significance, 95% CIs showed that average accuracy for both Sequence A and Sequence B remained around (and not differed significantly from) the 50% chance level; accuracy for Sequence A: *CI95%* [47.310, 51.272], accuracy for Sequence B: *CI95%* [48.628, 53.624]. No other main effect or interaction reached significance, all *p*s > .271, all *η_p_^2^* < .037, suggesting that the groups did not differ in their average accuracy, and that the effect of Sequence on accuracy was similar across groups.

The low performance on this measure could indicate at least two things: first, it may indicate that in spite of cueing the pattern trials under explicit task conditions, the knowledge of statistical structure (the relative frequency of different transitions) remained implicit for the participants. Second, participants may have had a knowledge about some triplets being more frequent than others, but they may have not been able to tell whether a particular transition was frequent during the Learning Phase (Sequence A) or the Rewiring Phase (Sequence B). Thus, low performance might have been caused by participants sorting high frequency trials as such, but not being able to correctly differentiate between Sequence A and Sequence B. This possibility should not be ignored considering that participants – on average – sorted 55.757% as high frequency trials, although in reality only 25% of them were truly high frequency (combined over the Learning and Rewiring Phases).

If a participant knew that a particular transition was frequent during one of the phases, but classified it as high frequency for the wrong sequence (e.g. classified it as being high frequency during Sequence A while in reality it was high frequency during Sequence B), this classification appears as a false alarm reducing overall accuracy scores. So we were interested in how many of such false alarms could be detected; we rerun the previously described ANOVA, but this time the dependent variable was this false alarm rate (correct classification of a transition but for the wrong sequence). The overall rate of such errors was 18.296% (*SEM* = 0.325, *CI95%* [17.647, 18.946]), thus not significantly different from chance level (18.75%). However, we found a significant main effect of **GROUP**, *F*(2, 69) = 3.946, *MSE* = 15.027, *p* = .024, *η_p_^2^* = 0.103. Post hoc tests revealed that the percentage of such errors was significantly lower in the case of the Implicit-Explicit group than in the case of the Implicit-Implicit group (*p* = .022, *d* = .829). The Explicit-Explicit group’s false alarm rate was between these two, not significantly different from either (both *p* > .312, *d* < .480). This result suggests that the interference was highest in the Implicit-Implicit group, and lowest in the Implicit-Explicit group. In spite of these differences, none of the groups showed more false alarms than expected by chance, as the 95% CIs included the 18.75 value.

In addition, there was a trend towards a main effect of **SEQUENCE**, *F*(1, 69) = 3.438, *MSE* = 17.459, p = .068, *η_p_^2^* = .047, as false alarms were more numerous in the case of Sequence A than for Sequence B (*d* = .323). In other terms, retroactive interference was higher than proactive interference, possibly reflecting that knowledge for Sequence A might become partly overwritten by knowledge for Sequence B. But again, despite these differences, false alarm rates remained around chance level (and did not differ from it significantly, as the 95% CIs included the 18.75 value). The interaction of **SEQUENCE x GROUP** was not significant, *p* = .834, *η_p_^2^* = .005, indicating that the previously described main effect of sequence was similar across groups.

In summary, the analysis of false alarms in the triplet sorting task shows that the interference caused by learning two, partly overlapping sequences was highest in the Implicit-Implicit group. This result suggests that the explicit cues indeed can help differentiate between the two sequences and use the acquired knowledge more appropriately in the relevant context.

# 3. REFERENCES

1 Howard, J. H., Jr. & Howard, D. V. Age differences in implicit learning of higher-order dependencies in serial patterns. *Psychol. Aging* **12**, 634-656, doi:10.1037/0882-7974.12.4.634 (1997).

2 Nemeth, D., Janacsek, K. & Fiser, J. Age-dependent and coordinated shift in performance between implicit and explicit skill learning. *Front. Comput. Neurosci.* **7**, doi:10.3389/fncom.2013.00147 (2013).

3 Jacoby, L. L. A process dissociation framework: Separating automatic from intentional uses of memory. *Journal of Memory and Language* **30**, 513-541 (1991).

4 Destrebecqz, A. & Cleeremans, A. Can sequence learning be implicit? New evidence with the process dissociation procedure. *Psychonomic Bulletin & Review* **8**, 343-350 (2001).

5 Song, S., Howard, J. H., Jr. & Howard, D. V. Perceptual sequence learning in a serial reaction time task. *Exp. Brain Res.* **189**, 145-158 (2008).

6 Fu, Q., Dienes, Z. & Fu, X. Can unconscious knowledge allow control in sequence learning? *Conscious. Cogn.* **19**, 462-474 (2010).

7 Gaillard, V., Cleeremans, A. & Destrebecqz, A. Dissociating conscious and unconscious learning with objective and subjective measures. *Clin. EEG Neurosci.*, 1550059413516757 (2014).
